# Decreased cobalamin sensitivity and biological aging acceleration in the general population

**DOI:** 10.1016/j.jnha.2024.100262

**Published:** 2024-05-20

**Authors:** Fan Tang, Hongbin Qiu, Yan Liu, Junchen Guo, Zheming Huang, Shaohong Fang, Yiying Zhang, Shanjie Wang

**Affiliations:** a Department of Cardiology, Second Affiliated Hospital of Harbin Medical University, Harbin, China; b The Key Laboratory of Myocardial Ischemia, Harbin Medical University, Ministry of Education, National Key Laboratory of Frigid Zone Cardiovascular Diseases, Harbin, China; c Department of Epidemiology and Biostatistics, School of Public Health, Jiamusi University, Jiamusi, China

**Keywords:** Biological aging, KDM age, Phenotypic age, Cobalamin, Methylmalonic acid, Homocysteine

## Abstract

**Background:**

The evidence on the association between cobalamin (Cbl) and aging or relevant outcomes is limited and controversial. We aimed to investigate the relationships between cobalamin intake- and function-related biomarkers and biological aging.

**Methods:**

The study encompassed 22,812 participants aged 20 years and older from the National Health and Nutrition Examination Survey. A panel of biomarkers or algorithms was used to assess biological aging, including Klemera–Doubal Age Acceleration (KDMAccel), Phenotypic age acceleration (PhenoAgeAccel), telomere length, α-Klotho, and PhenoAge advancement. Weighted generalized linear regression analysis was used to assess the associations between cobalamin-intake biomarkers (serum cobalamin, cobalamin intake from food, cobalamin supplement use, serum methylmalonic acid [MMA], and homocysteine [Hcy]) and function-related biomarkers (functional cobalamin deficiency and cobalamin insensitivity index).

**Results:**

Among the 22,812 individuals, the weighted mean (SE) age was 48.3 (0.2) years and 48.0% were males. Unexpectedly, serum and dietary cobalamin as well as serum MMA and Hcy levels were positively associated with most indicators of biological aging. Cobalamin sensitivity was assessed by the combination of binary Cbl_low/high_ and MMA_low/high_ or Hcy_low/high_ (cutoff values: 400 pg/mL for cobalamin, 250 nmol/L for MMA, and 12.1 μmol/l for Hcy) and a newly constructed cobalamin insensitivity index (based on the multiplicative term of serum cobalamin and serum MMA or Hcy). The multivariable-adjusted β (95%CIs) of KDMAccel in the MMA_low_Cbl_low_, MMA_low_Cbl_high_, MMA_high_Cbl_low_, and MMA_high_Cbl_high_ groups were reference, 0.27 (0.03 to 0.51), 0.85 (0.41 to 1.29), and 7.97 years (5.77 to 10.17) respectively, which were consistent for the combination of serum Hcy and cobalamin. Both cobalamin insensitivity indices were robustly associated with biological aging acceleration in a dose-response pattern (each p < 0.001).

**Conclusions:**

Decreased cobalamin sensitivity but not cobalamin insufficiency might be associated with biological aging acceleration. Further studies would improve understanding of the underlying mechanisms between decreased cobalamin sensitivity and biological aging acceleration.

## 1 Introduction

At present, an estimated 900 million population worldwide are aged 65 or older, and this demographic is projected to surpass 2 billion (16%) by 2050 [1], posing a formidable challenge to both healthspan and lifespan. One-quarter of the global cost of the disease is borne by the older population, meaning that maintaining one’s healthspan is a public health priority [2]. Biological aging, which refers to functional decline across multiple tissues and organs, has proven to be a superior predictor of chronological age [3]. Numerous biomarkers of biological aging, including leukocyte telomere length [4] and α-klotho [5], have been identified. In addition, algorithms integrating information across epigenetic, proteomic, and metabolomic profiles and other molecular levels of analysis, such as the Klemera–Doubal method biological age (KDM Age) [6] and Phenotypic Age(PhenoAge) [7], have been developed.

Evidence from a population-based longitudinal study indicated that cobalamin (Cbl) and total homocysteine (Hcy) concentrations might be linked to brain aging [8]. However, several cohort studies reported that there was no significant association between cobalamin levels and frailty, a syndrome related to aging, in older adults [[9], [10], [11]]. Overall, the benefits of cobalamin supplementation in aging diseases were unclear. In contrast, methylmalonic acid (MMA) was found to be associated with phenotypic age acceleration [12]. According to the Singapore Longitudinal Aging Study with 238 participants, blood MMA levels were significantly greater in older subjects with aging and frailty conditions than in younger controls [13], but this study had a smaller sample size.

Furthermore, functional cobalamin deficiency, characterized by elevated metabolites even when cobalamin levels fall within the normal reference range, affects 7–30% of various elderly populations [14]. This condition is linked to cognitive decline, anemia, and neurological disorders [14]. Functional cobalamin deficiency, which arises from cobalamin oxidation and inactivation, may progress more swiftly than typical cobalamin deficiency because it does not deplete the body's reserves of cobalamin. Even higher and more frequent doses of cobalamin have limited effects, indicating reduced sensitivity to cobalamin treatment [15,16]. The heterogeneity of cobalamin sensitivity may explain the inconsistent findings regarding cobalamin levels and adverse outcomes [[17], [18], [19], [20]]. In addition to cobalamin supplementation, decreased sensitivity to cobalamin should be of greater concern for promoting health. To address these knowledge gaps, we comprehensively investigated the associations of cobalamin-intake biomarkers (serum cobalamin, cobalamin intake from food, cobalamin supplement use, serum MMA, and serum homocysteine [Hcy]) and function-related biomarkers (functional cobalamin deficiency and the cobalamin insensitivity index) with biological aging in a nationally representative sample of US adults.

## 2 Methods

### 2.1 Study population

**Fig. 1:**
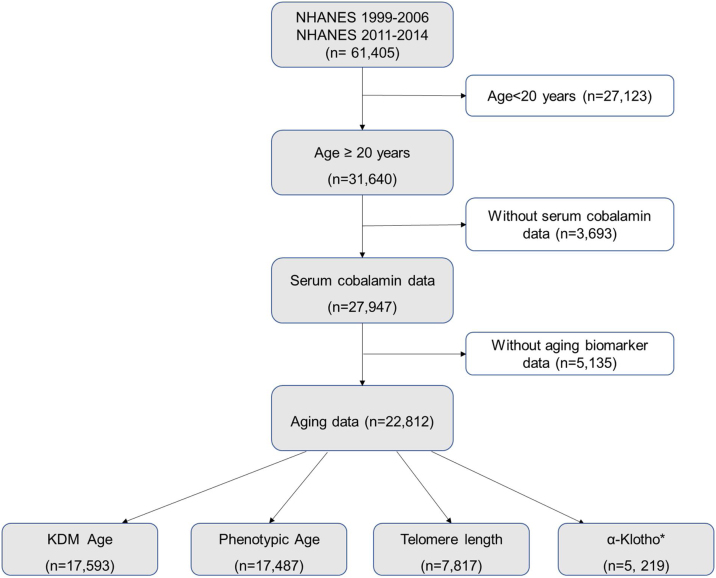
Flow of study. *Investigation of α-Klotho is conducted exclusively in males and females aged 40–79 years.

All data were obtained from the National Health and Nutrition Examination Survey (NHANES) conducted by the US Centers for Disease Control and Prevention (CDC). As previously described [18,21], the NHANES is a sustained, national, stratified, multistage probability survey crafted to assess the health status of a noninstitutionalized civilian population of all age groups. In this study, we enrolled 31,640 participants aged ≥20 in six NHANES survey cycles (1999–2000, 2001–2002, 2003–2004, 2005–2006, 2011–2012, and 2013–2014). We excluded individuals without data on serum cobalamin (n = 3,693) and aging biomarkers (n = 5,135). Our final analytic dataset included 22,812 participants (Fig. 1). The participants were divided into four subgroups: KDM Age (N = 17,593), Phenotypic Age (N = 17,487), Telomere Length (N = 7,817), and α-Klotho (N = 5,219). The study protocol received approval from the ethics review board at the National Center for Health Statistics, and informed consent was obtained from all participants.

### 2.2 Study exposure: Cobalamin intake- and function-related biomarkers

All specimens were analyzed at the NHANES central laboratory. Between 1999 and 2006, serum cobalamin concentrations were assessed using the"Quantaphase II Cobalamin" radioassay kit from Bio-Rad Laboratories. From 2011 to 2014, serum cobalamin levels were quantified utilizing a Roche electrochemiluminescence immunoassay. The two analytical methods demonstrated comparable coefficients of variation (<5%) and similar detection limits (20−30 pg/mL).To ensure data consistency, all concentrations were converted to Bio-Rad values using the following formula for serum cobalamin Bio-Rad in pg/mL = 10^(1.03 × log10 (Roche cobalamin) − 0.14), taking into account errors in both variables as determined by Deming regression analysis [18].

Dietary recalls in the NHANES are conducted by trained interviewers from the Mobile Examination Center using a standardized set of measurement guidelines to assist participants in reporting the volume and size of food consumed [20]. Supplement use was assessed through self-reported supplement intake in the past 30 days by participants. Participants reporting supplement usage were asked to provide information on the name of the supplement, frequency of use, and typical dosage. We extracted information related to the use of cobalamin supplements.

Serum MMA levels were determined through the application of gas chromatography-mass spectrometry techniques from 1999 to 2004 and liquid chromatography-tandem mass spectrometry method from 2011 to 2014. Based on an internal comparison, liquid chromatography-tandem mass spectrometry exhibited a high degree of correlation and robust concurrence with measurements procured through gas chromatography-mass spectrometry [22].

Total plasma Hcy levels were detected in blood samples from NHANES 1999–2006 participants using the Abbott homocysteine assay. The method for assessing homocysteine was transitioned from Abbott IMX to the Abbott AxSym method in 2002. Next, a crossover study was performed between the two methods and the results revealed a superb correlation (n = 361, r2 = 0.9817) [23].

Based on previous studies, functional cobalamin deficiency (characterized by impaired response to cobalamin treatment) was defined as a serum MMA level greater than 250 nmol/l, a serum Hcy level greater than 12.1 μmol/l, and a serum cobalamin level greater than 400 pg/mL [14]. The study population was classified based on the combination of serum cobalamin and MMA levels to assess cobalamin sensitivity: MMA_low_Cbl_low_ (MMA ≤ 250 nmol/L, cobalamin ≤ 400 pg/mL), MMA_low_Cbl_high_ (MMA ≤ 250 nmol/L, cobalamin>400 pg/mL), MMA_high_Cbl_low_ (MMA > 250 nmol/L, cobalamin≤400 pg/mL), and MMA_high_Cbl_high_ (MMA > 250 nmol/L, cobalamin>400 pg/mL). Additionally, the study population was also categorized into four distinct groups based on the combination of serum cobalamin and Hcy levels: Hcy_low_Cbl_low_ (Hcy≤12.1 μmol/l, and cobalamin≤400 pg/mL), Hcy_low_Cbl_high_ (Hcy≤ 12.1 μmol/l, and cobalamin>400 pg/mL), Hcy_high_Cbl_low_ (Hcy>12.1 μmol/l, and cobalamin≤400 pg/mL), and Hcy_high_Cbl_high_ (Hcy>12.1 μmol/l, and cobalamin>400 pg/mL). Considering the qualitative nature of the combination of binary serum cobalamin and MMA/Hcy, we developed the cobalamin insensitivity index as a surrogate method to quantify the extent of the decreased response to cobalamin, calculated as MMA*Cbl/100 and Hcy*Cbl/100.

### 2.3 Study outcomes: Biological aging-related indicators

We quantified biological aging using two methods: Klemera–Doubal method of biological age (KDM BioAge) and Phenotypic Age. Both have been validated in multiethnic cohorts of older adults to predict disease, disability, and mortality [[24], [25], [26]]. The BioAge R package allows us to customize the KDM and PhenoAge algorithms with specific biomarkers and apply them to new datasets [27]. KDM BioAge is based on eight biomarkers: Ln-C-reactive protein (CRP), serum creatinine, glycosylated hemoglobin, serum albumin, serum total cholesterol, serum urea nitrogen, serum alkaline phosphatase, and systolic blood pressure [28,29]. Phenotypic Age, on the other hand, includes nine aging-related variables: chronological age, albumin, creatinine, glucose, CRP, lymphocyte percentage, mean cell volume, red blood cell distribution width, alkaline phosphatase, and white blood cell count [[27], [28], [29]].

To assess biological aging acceleration, we calculated the residuals by regressing chronological age on biological age, referred to as 'KDMAccel' and 'PhenoAgeAccel' [28]. For detailed calculation formulas for biological aging and aging acceleration, please refer to the Supplementary methods. For our analysis, we computed PhenoAge advancement as the difference between the predicted biological age and the chronological age [27]. PhenoAge advancement was subsequently standardized to exhibit a mean of 0 and a standard deviation of 1. A positive value for PhenoAge advancement denotes an escalated stage of biological aging and increased susceptibility to disease and mortality [27]. Conversely, a negative PhenoAge advancement signifies a decelerated biological aging process. In our study, we also observed a strong correlation between chronological age and KDM Age (R = 0.97) and phenotypic age (R = 0.92) (Figure S1).

Telomere length was ascertained using quantitative polymerase chain reaction (PCR), allowing for the evaluation of telomere length relative to that of standard reference DNA (T/S ratio) [30]. In the majority of cells, telomeres undergo shortening with each cell cycle [31]. When telomeres become very short, cells enter senescence, cell cycle arrest, or undergo apoptosis [31], it is considered a marker of biological aging.

Serum α-klotho levels in frozen samples from individuals aged 40–79 years, collected during NHANES 2011–2014, were analyzed in 2019–2020. ELISA kits from IBL International, Japan, were used for analysis, with samples stored at −80 °C until they were attached for analysis. A total of 114 samples from healthy donors were assessed, yielding a reference range of 285.8 to 1638.6 pg/mL (mean = 698.0 pg/mL) [32]. Mice deficient in α-Klotho display premature aging phenotypes [33]. Decreased levels of serum α-Klotho have been validated to be robustly associated with age [34] and thus considered a hallmark of aging.

### 2.4 Other variables

Sociodemographic variables encompassed age, sex (male/female), race or ethnicity (Mexican-American, other Hispanic, non-Hispanic white, non-Hispanic black, other race), education level (less than high school, high school graduate, more than high school), marital status (married/cohabitating, divorced/widowed/separated, never married), and poverty income ratio (<1.3, 1.3–3.5, >3.5). Body mass index (BMI) was categorized as normal (<25.0 kg/m²), overweight (25.0–29.9 kg/m²), or obese (≥30.0 kg/m²). Lifestyle variables included smoking status (never, former, current), heavy alcohol consumption (male ≥20 g/day, female ≥10 g/day), and physical activity (less, moderate, vigorous). Health-related factors consisted of cardiovascular disease (CVD), type 2 diabetes, and hypertension. According to the previous NHANES report [22], CVD was defined as a self-reported history of coronary heart disease, heart failure, or stroke, type 2 diabetes was defined as self-reported type 2 diabetes, and hypertension was defined as a mean baseline systolic blood pressure of 140 mmHg or more or diastolic blood pressure of 90 mmHg or more and the use of antihypertensive medication.

### 2.5 Statistical analysis

We performed all analyses following the NHANES guidelines for analyzing datasets, as reported previously [19]. To address the multistage sampling design and ensure national representation, we employed masked variance in the main sampling unit, pseudostates, and sampling weights. Weighted means (standard errors [SE] and percentages are used for numerical and categorical variables. Owing to their skewed distribution and broad range, log2-transformed serum and dietary cobalamin, serum MMA, and serum Hcy were used for correlation analysis.

Quadratic fitting lines and heat maps were used to flexibly evaluate the associations of cobalamin and its deficiency biomarkers with biological aging. Regression coefficients (β) and 95% confidence intervals (CIs) were assessed using survey-weighted multiple linear regression models to investigate the associations between serum cobalamin, cobalamin intake from food, cobalamin supplementation (use vs. nonuse), serum MMA levels, serum Hcy levels, and cobalamin sensitivity (functional cobalamin deficiency, newly constructed cobalamin insensitivity index), and biological aging indicators (KDM Age, Phenotypic age, KDMAccel, PhenoAgeAccel, telomere length, α-Klotho). In addition, weighted multivariate logistic regression was used to estimate odds ratios (ORs) and corresponding 95% CIs to assess the association of cobalamin and its deficiency biomarkers with PhenoAge advancement. Two models were constructed: Model 1 was adjusted for age, sex, and race/ethnicity. Model 2 expanded on Model 1 to include adjustments for education level, marital status, household income and poverty rate, BMI, physical activity, alcohol use, smoking, type 2 diabetes, hypertension, and CVD.

Additional analyses were conducted to circumvent reverse causation and enhance the stability of the results. In the four periods of the NHANES (1999–2000, 2001–2002, 2003–2004, and 2005–2006), participants aged 85 and over 85 years were recorded as 85 years old. However, in the NHANES 2011–2012 and 2013–2014, participants aged 80 and over 80 years old were recorded as 80 years old. Consequently, the chronological age of participants older than 80 years is unknown. Thus, we opted to exclude participants over 80 years old from the analyses. Finally, sex and age subgroups were analyzed.

Since the amount of missing data in the covariates was small (<5%), we used multivariate imputation by chain equations (MICE) to handle the missing data [20]. The statistical analyses were performed using STATA (version 15.1, Stata Corp LP, Texas, USA), with *p*-value of less than 0.05 indicating statistical significance.

## 3 Results

### 3.1 Participant characteristics

The study involved 22,812 adults aged 20 years or older. The baseline characteristics of the study cohort are illustrated in Table 1. The mean age was 48.3 years, and 48.0% were male. Approximately 39.1% of the study population used cobalamin dietary supplements. The average daily intake of cobalamin from food was 5.2 μg. The mean concentrations of serum cobalamin, MMA, and Hcy were 363.6 pg/mL, 163.8 nmol/L, and 8.6 μmol/L, respectively. The weighted means of the biological aging markers (KDM Age: 45.7 years, Phenotypic age: 41.5 years, telomere length: 1.1 T/S ratio, and α-Klotho:862.4 pg/mL) exhibited minimal changes from the 1999–2006 cycle to the 2011–2014 cycle (Table S1).

**Table 1:** Baseline characteristics of participants in NHANES 1999-2014.

Variables	Mean ± SE or n (%)
Age, years	48.3 ± 0.2
Sex, %	
Male	11000 (48.0)
Race/ethnicity, %	
Mexican American	4548 (7.2)
Other Hispanic	1274 (5.2)
Non-Hispanic White	11012 (72.2)
Non-Hispanic Black	4582 (10.3)
Other Race	1396 (5.3)
Education, %	
Less than high school	6787 (18.7)
High school	5273 (24.6)
More than high school	10752 (56.7)
Marital status, %	
Married/Living with a partner	13164 (60.7)
Widowed/Divorced/Separated	4496 (20.3)
Never Married	5152 (19.0)
Poverty income ratio, %	
<1.30	7807 (24.9)
1.31−3.5	7943 (33.5)
>3.5	7062 (41.6)
Body mass index, kg/m^2^	
>25	6695 (31.3)
25 to 30	7883 (33.9)
≥30	8234 (34.8)
Smoking status, %	
Never	11884 (51.0)
Former	6122 (25.9)
Current	4806 (23.1)
Physical activity, %	
Less	10496 (38.9)
Moderate	6580 (30.2)
Vigorous	5736 (30.9)
Heavy drinking, %	1456 (8.1)
Serum cobalamin, pg/mL	401.7 ± 5.9
Cobalamin intake from foods, ug/day	5.2 ± 0.1
Cobalamin supplements, %	6767 (39.1)
Methylmalonic acid, nmol/L	163.8 ± 1.6
Homocysteine, μmol/l	8.6 ± 0.1
Telomere Length, T/S ratio	1.1 ± 0.1
α-Klotho^a^, pg/mL	862.4 ± 7.1
KDM Age, years	45.7 ± 0.3
Phenotypic Age, years	41.2 ± 0.3
Type 2 Diabetes, %	3074 (9.6)
Cardiovascular diseases, %	2636 (9.0)
Hypertension, %	9899 (38.1)

Percentages, means, and standard errors are adjusted for NHANES sampling weights. The observed numbers for categorical variables were unweighted.

Abbreviations: SE, standard errors; Heavy drinking (male ≥20 g/day, female ≥10 g/day); KDM, Klemera–Doubal method.

a Investigation of α-Klotho is conducted exclusively in males and females aged 40–79 years.

### 3.2 The relationship between cobalamin and related biomarkers and biological aging

**Fig. 2:**
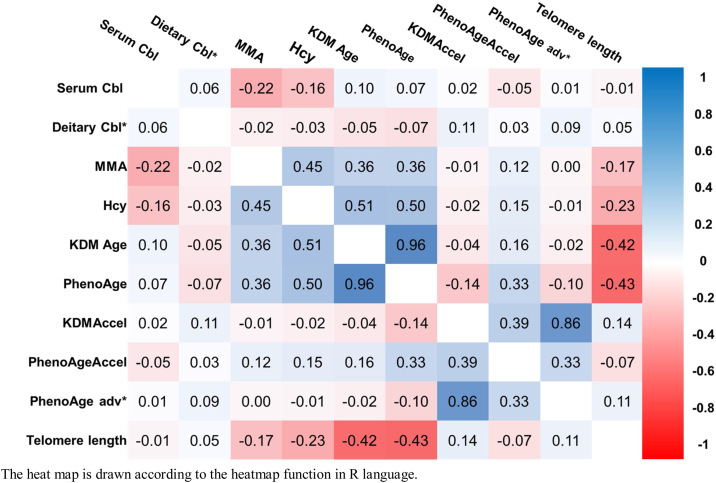
The correlation matrix of cobalamin and its biomarkers and biological aging-related indicators in NHANES. The heat map is drawn according to the heatmap function in R language. Abbreviations: Cbl, cobalamin; Dietary Cbl: cobalamin intake from food; MMA, methylmalonic acid; Hcy, homocysteine; KDM, Klemera–Doubal method; PhenoAge, Phenotypic Age; KDM Accel, KDM Acceleration; PhenoAgeAccel, Phenotypic Age Acceleration; PhenoAge adv*: Phenotypic Age advancement

**Fig. 3:**
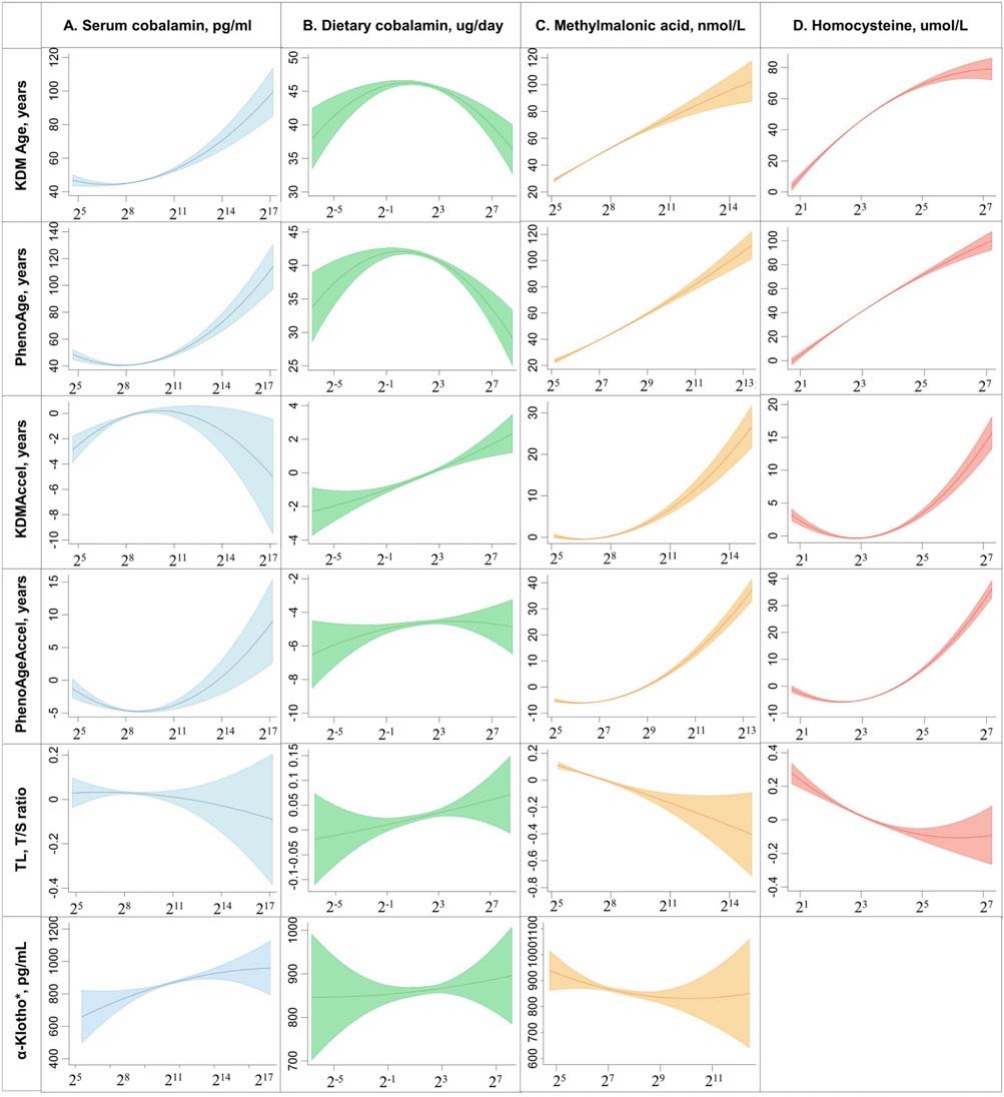
Fitting plot of cobalamin and its biomarkers and biological aging-related indicators. The graph represents the linearity of the response association of log-transformed serum cobalamin, log-transformed cobalamin intake from food, log-transformed serum methylmalonic acid, and log-transformed serum homocysteine with biological aging-related indicators. The blue-, green-, orange-, red-shaded region shows the 95% confidence intervals (CIs) around the regression line; Abbreviations: KDM, Klemera–Doubal method; KDM Accel, KDM Age Acceleration; PhenoageAccel, Phenotypic Age Acceleration; TL, Telomere Length; Dietary cobalamin: Cobalamin intake from food; *Investigation of α-Klotho is conducted exclusively in males and females aged 40–79 years

The correlation matrix between cobalamin and its deficiency biomarkers with biological aging-related indicators is illustrated in Fig. 2. Serum and dietary cobalamin, serum MMA, and Hcy were positively correlated with KDM age, phenotypic age, KDMAccel, PhenoAgeAccel, and PhenoAge advancement. The maximum correlation coefficient among these variables was 0.51. Linear regression fit between cobalamin, its deficiency biomarkers, and biological aging (Fig. 3). Serum MMA and Hcy showed a positive correlation with biological aging-related indicators, including the KDM age, phenotypic age, KDMAccel, and PhenoAgeAccel, and a negative correlation with telomere length and α-Klotho. When the samples were segmented by normal cobalamin levels (≥339 pg/mL [≥250 pmol/L]), we found that cobalamin-related biomarkers exhibited weak correlations with the single biomarkers included in the algorithms of biological aging (Table S2-S3).

**Fig. 4:**
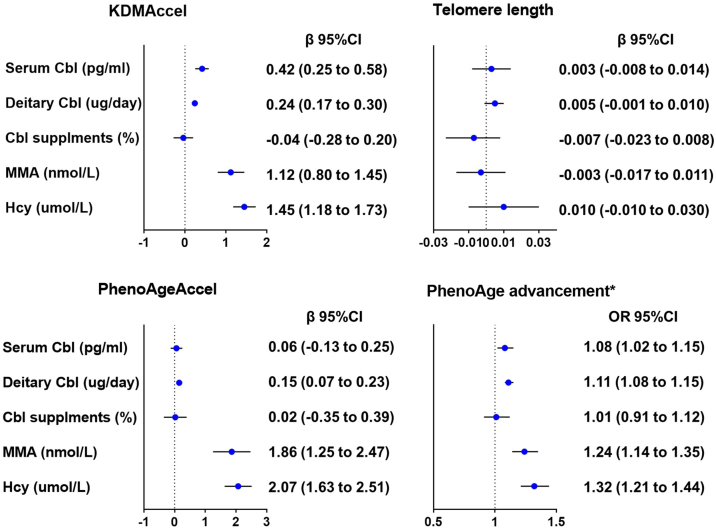
The relationship between cobalamin and its biomarkers and biological aging. Abbreviations: Cbl, cobalamin; Dietary Cbl: Cobalamin intake from food; KDM, Klemera–Doubal method; KDM Accel, KDM Acceleration; PhenoageAccel, Phenotypic Age Acceleration; PhenoAge advancement, Phenotypic Age advancement. Dots (centers of error bars): Point estimate; Error bar: 95% confidence limits; Dash line: Reference line. Data were adjusted for survey weights of NHANES. *PhenoAge advancement is a binary variable, the results are shown as OR, 95%CI. Adjusted for Model 2: age (continuous), sex (female, male), race/ ethnicity (Mexican-American, other Hispanic, non-Hispanic white, non-Hispanic black, other race), education level (less than high school, high school, more than high school), marital status (married/cohabitating, divorced/widowed/separated, never married), Poverty income ratio (<1.3, 1.3–3.5, >3.5). body mass index (<25.0 kg/m²), 25.0–29.9 kg/m², ≥30.0 kg/m²), smoking status (never, former, current), heavy alcohol consumption (male ≥20 g/day, female ≥10 g/day), physical activity (less, moderate, vigorous), Type 2 Diabetes (Yes/no), hypertension (Yes/no) and cardiovascular diseases (Yes/no).

The relationship between serum cobalamin and biological aging (Fig. 4 and Table S4). There was a positive association between serum cobalamin and KDM Accel, and α-Klotho in all three linear regression models. In the fully adjusted regression model, for each doubling of serum cobalamin, KDM Accel increased by 0.42 years (95%CI: 0.25 to 0.58, P < 0.001), and α-Kloth increased by 23.18 pg/mL (95%CI: 12.56–33.80, P < 0.001). The doubling of serum cobalamin was associated with higher odds of PhenoAge advancement (OR = 1.08, 95%CI: 1.02 to 1.15).

The relationship between cobalamin intake from food and biological aging (Fig. 4 and Table S4). In the fully adjusted regression model, each doubling of cobalamin intake from food was associated with an increase of 0.24 years in KDMAccel (95%CI: 0.17 to 0.30, P < 0.001), as well as an increase of 0.15 years in PhenoAgeAccel (95%CI: 0.07 to 0.23, P < 0.001). Cobalamin intake from food was significantly associated with PhenoAge advancement, after adjustment for Model 2 (OR = 1.11, 95%CI: 1.08 to1.15).

In addition, considering the potential effect of the source of cobalamin intake, compared with non-users (Fig. 4 and Table S4), Cobalamin supplement use did not significantly delay biological aging (KDMAccel (P = 0.72), Phenotypic Age and PhenoAgeAccel (P = 0.91), telomere length (P = 0.352), α-Klotho (P = 0.93), and PhenoAge advancement (P = 0.88).

In contrast, higher serum MMA and Hcy were significantly associated with biological aging-related indicators (Fig. 4 and Table S5). After adjusting for Model 2, for each doubling of serum MMA, KDMAccel increased by 1.12 years (95% CI: 0.8–1.45, P < 0.001), PhenoAgeAccel increased by 1.86 years (95%CI: 1.25–2.47, P < 0.001), and α-Klotho concentration decreased by 18.37 pg/mL (95%CI: -32.55 to -4.20, P = 0.01). With each doubling of the serum Hcy concentration, the KDMAccel increased by 1.45 years (95% CI:1.18 to 1.73, P < 0.001), and the PhenoAgeAccel increased by 2.07 years (95% CI:1.63 to 2.51, P < 0.001).

### 3.3 The relationship between functional cobalamin deficiency and biological aging

**Fig. 5:**
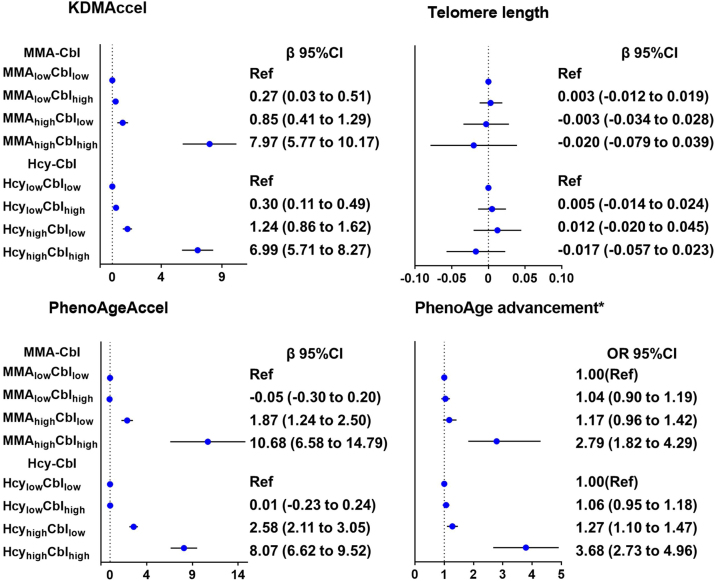
The relationship between functional cobalamin deficiency and biological aging. Abbreviations: Cbl, cobalamin; KDM, Klemera–Doubal method; KDMAccel, KDM Age Acceleration; PhenoageAccel, Phenotypic Age Acceleration; PhenoAge advancement, Phenotypic Age advancement; MMA, Methylmalonic acid; Hcy, Homocysteine. Dots (centers of error bars): Point estimate; Error bar: 95% confidence limits; Dash line: Reference line. Data were adjusted for survey weights of NHANES. *PhenoAge advancement is a binary variable, the results are shown as OR, 95%CI. Both indicators were categorized into high vs. low levels and combined into four groups according to the prespecified cutoff values (MMA > 250 nmol/L or Hcy>12.1 μmol/l and cobalamin >400 pg/mL). Adjusted for Model 2: age (continuous), sex (female, male), race/ ethnicity (Mexican-American, other Hispanic, non-Hispanic white, non-Hispanic black, other race), education level (less than high school, high school, more than high school), marital status (married/cohabitating, divorced/widowed/separated, never married), Poverty income ratio (<1.3, 1.3–3.5, >3.5). Body mass index (<25.0 kg/m²), 25.0–29.9 kg/m², ≥30.0 kg/m²), smoking status (never, former, current), heavy alcohol consumption (male ≥20 g/day, female ≥10 g/day), physical activity (less, moderate, vigorous), Type 2 Diabetes (Yes/no), hypertension (Yes/no) and cardiovascular diseases (Yes/no).

The relationship between functional cobalamin deficiency and biological aging revealed that participants in the MMA_high_Cbl_high_ group, as well as the Hcy_high_Cbl_high_ group, exhibited faster aging acceleration (Fig. 5 and Table S6-S7). After adjustment for all covariates, compared with participants in the MMA_low_Cbl_low_ group, participants in the MMA_high_Cbl_low_ group had an increase of 0.85 years in KDMAccel (95%CI:0.41 to 1.29, P < 0.001), and 1.87 years in PhenoAgeAccel (95%CI:1.24 to 2.50, P < 0.001), and the multivariable-adjusted OR and 95%CI for PhenoAge advancement were 1.17 (0.96–1.42). The participants in the MMA_high_Cbl_high_ group had an increase of 7.97 years in KDMAccel (95%CI: 5.77–10.17, P < 0.001), and 10.68 years in PhenoAgeAccel (95%CI: 6.58 14.79, P < 0.001), and the multivariable-adjusted OR and 95%CI for PhenoAge advancement were 2.79 (1.82–4.29).

Compared with participants in the Hcy_low_Cbl_low_ group, participants in the Hcy_high_Cbl_low_ group had an increase of 1.24 years in KDMAccel (95% CI:0.86 to 1.62, P < 0.001), and 2.58 years in PhenoAgeAccel (95%CI:2.11 to 3.05, P < 0.001), the multivariable-adjusted OR and 95%CI for PhenoAge advancement were 1.27 (1.10 to 1.47. Participants in the Hcy_high_Cbl_high_ group had increased KDMAccel by 6.99 years (95% CI: 5.71–8.27, P < 0.001) and PhenoAgeAccel by 8.07 years (95%CI:6.62 to 9.52, P < 0.001), and the multivariable-adjusted OR (95%CI) for PhenoAge advancement was 3.68 (2.73–4.96).

**Fig. 6:**
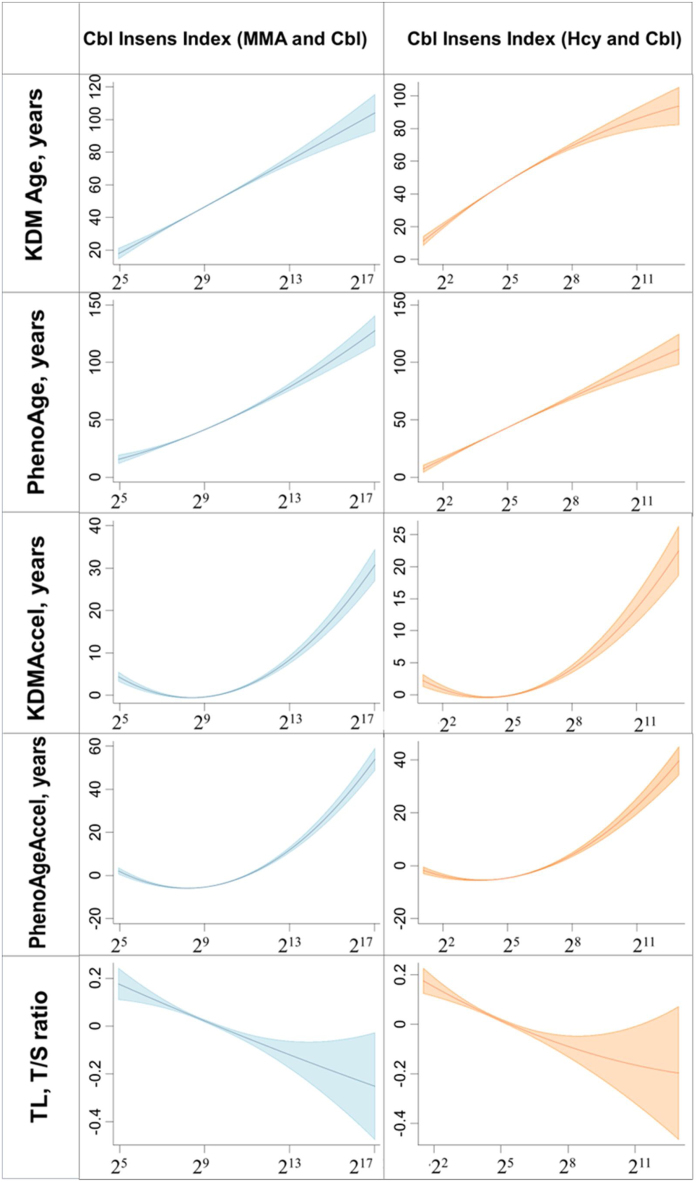
Fitting plot of cobalamin insensitivity index and biological aging-related indicators. The graph represents the linearity of the response association of Log-transformed the cobalamin insensitivity index with biological aging-related indicators. The blue-, green-, orange-shaded region shows the 95% confidence intervals (CIs) around the regression line. Abbreviations: KDM, Klemera–Doubal method; KDM Accel, KDM Acceleration; PhenoAgeAccel, Phenotypic Age Acceleration, TL, Telomere Length; Cbl, cobalamin; Cbl Insens Index, Cbl Insensitivity Index; MMA, Methylmalonic acid; Hcy, Homocysteine. Cobalamin insensitivity index estimated by Methylmalonic Acid or Homocysteine and cobalamin. Cbl Insens Index (MMA and Cbl): a multiplicative term by Methylmalonic Acid and cobalamin divided by 100. Cbl Insens Index (Hcy and Cbl): a multiplicative term by Homocysteine and cobalamin divided by 100.

To examine cobalamin sensitivity, we constructed a new cobalamin insensitivity index (Fig. 6 and Table S8–S9). With each doubling of the cobalamin insensitivity index based on MMA, the biological aging-related indicator KDMAccel increased by 1.10 years (95% CI: 0.84–1.35, P < 0.001), PhenoAgeAccel increased by 1.39 years (95% CI: 0.89–1.90, P < 0.001), and the multivariable-adjusted OR and 95%CI for PhenoAge advancement were 1.21 (1.13–1.30). For each doubling of the cobalamin insensitivity index based on Hcy, KDMAccel increased by 0.99 years (95% CI: 0.82–1.15, P < 0.001), PhenoAgeAccel increased by 0.88 years (95%CI: 0.68–1.08, P < 0.001), and the multivariable-adjusted OR and 95%CI for PhenoAge advancement were 1.21 (1.14–1.29). In addition, the associations between both the cobalamin insensitivity index and biological aging remained significant in the stratification analyses stratified by cobalamin dietary supplement use (yes/no) and cobalamin intake from food (median value) (Table S10-S11).

### 3.4 Additional analysis

When we excluded the elderly population (aged >80) (Table S12), aging acceleration was still observed with increasing serum and dietary cobalamin, serum MMA, and Hcy. There was no significant association between cobalamin supplement use and biological aging compared to non-users. Compared with participants in MMA_low_Cbl_low_ group, participants in MMA_high_Cbl_high_ group have a faster biological aging acceleration, and compared with participants in Hcy_low_Cbl_low_ group, participants in Hcy_high_Cbl_high_ group have a faster biological aging acceleration.

To enhance the robustness of our research findings, we conducted subgroup analyses based on age and sex (Table S13-S14). Our results showed that MMA, Hcy, and functional cobalamin deficiency were significantly associated with biological aging-related indicators, including KDMAccel, PhenoageAccel, and PhenoAge advancement, among individuals over 60 years of age and women.

## 4 Discussion

In this extensive, population-based study comprising 22,812 representative participants from across the U.S, we calculated biological aging using a panel of aging-related indicators, including KDMAccel, PhenoAgeAccel, telomere length, α-Klotho, and PhenoAge advancement. Moreover, we investigated the relationship between serum cobalamin, its biomarkers, and biological aging. Our findings suggest that serum MMA or Hcy is associated with increased aging acceleration. Both the serum and dietary cobalamin levels were positively correlated with most indicators of biological aging. However, against expectations, cobalamin supplement use did not significantly impact biological aging when compared to non-users. Further analysis showed that this heterogeneity may be mainly attributed to functional cobalamin deficiency. Compared with participants in the MMA_low_Cbl_low_ group, the biological aging acceleration in the MMA_high_Cbl_high_ group in the KDMAccel increased by at least 7.97 times. Similarly, compared to participants in the Hcy_low_Cbl_low_ group, the biological aging acceleration of participants in the Hcy_high_Cbl_high_ group in KDMAccel increased by at least 6.99 times. In addition, lower cobalamin sensitivity is associated with multiple biological aging indicators.

Cobalamin levels are known to decrease with age. Numerous population surveys have recorded a high prevalence of cobalamin deficiency among older adults globally, such as in the United States, where 6% are deficient and more than 20% are borderline. These surveys indicate that older adults exhibit varying degrees of cobalamin deficiency and suboptimal status [35]. Poor cobalamin status is associated with several age-related chronic diseases [36]. However, despite widespread recommendations of cobalamin for the prevention of age-related diseases, the benefits derived from cobalamin interventions in the elderly have been limited. Placebo-controlled randomized clinical trials have revealed that cobalamin supplementation restores circulating cobalamin levels and direct biomarkers of cobalamin deficiency in some, but not all, older adults [37]. This largely indicates that participants are less responsive to cobalamin.

Our results indicate that MMA and Hcy are associated with biological aging. Similarly, another study identified elevated levels of circulating MMA as a potential biomarker of mitochondrial dysfunction, contributing to biological aging [12]. In comparison to cobalamin, MMA and Hcy are more likely to serve as markers of biological aging. However, it is important to note that our cross-sectional study has certain limitations, and further clinical research is necessary to validate these findings. In humans, serum α-klotho concentrations decrease with age [38]. In a cross-sectional study, a significant association was discovered between increased serum α-klotho concentrations and increased serum cobalamin concentrations [39], but the present study revealed that serum cobalamin was associated with serum α-klotho, suggesting a complex interrelationship between serum cobalamin and serum α-klotho. Telomere length is considered a biomarker of cellular aging; however, our study revealed that cobalamin-related biomarkers were not associated with telomere length. Similarly, in a subsample of 1715 women from the Nurses' Health Study [40] and another cross-sectional study of 798 men and women aged 55–79 years [41], no significant association was observed between cobalamin and telomere length.

Accumulating evidence points to a complex interplay between the process of aging and the metabolism and function of cobalamin [14]. A wealth of pathophysiological evidence from studies involving older adults and aged mice suggests a decline in the body's capacity to absorb cobalamin with age, accompanied by a concurrent reduction in its reabsorption by the kidneys [13]. In addition to the uptake process, cobalamin plays a significant role in numerous vital steps of MMA metabolism, including encompassing endocytosis and trafficking, release into cells via lysosomes, and activation within mitochondria [42]. Notably, disruptions in lysosomal and mitochondrial function frequently occur under chronic pathological conditions, which impede the intracellular bioavailability of cobalamin [43,44]. Clinical studies on high-dose cobalamin supplements have not concluded significant benefits [45,46]. This is consistent with the null association between cobalamin supplement use and biological aging indicators in our study. We recognize that the cross-sectional design of our study poses challenges in asserting causal inferences regarding the acceleration of aging with serum cobalamin and dietary cobalamin intake. This phenomenon could be explained by the correlation between the decrease in cobalamin reactivity and the acceleration of aging. A compensatory increase in cobalamin intake might be insufficient to confer benefits related to the age-associated decrease in cobalamin reactivity. Our study underscores the need for future investigations to further illuminate the pathophysiological mechanisms underpinning functional cobalamin deficiency and to elucidate the intrinsic correlation between functional cobalamin deficiency and the acceleration of aging.

The cobalamin insensitivity index was proposed in our previous studies [[17], [18], [19], [20]], and we believe that this term could more accurately reflect pathophysiological conditions than functional cobalamin deficiency. Lower cobalamin sensitivity was strongly associated with multiple biological aging indicators. Upon stratification based on the use of cobalamin supplements and the median cobalamin intake from food, we observed a significant correlation between the cobalamin insensitivity index and biological aging. This indicates that the relationship between the cobalamin insensitivity index and biological aging remains significant regardless of whether cobalamin is taken from food or cobalamin dietary supplements. However, due to the limitations of cross-sectional studies, we cannot obtain causal links. Although the underlying mechanisms of decreased cobalamin sensitivity have not been fully elucidated, oxidative stress and mitochondrial dysfunction appear to be significant characteristics [14,42,47]. Other biological studies on mitochondria may provide some insight into impaired cobalamin sensitivity. Cellular aging is a response initiated by acute or chronic damage in humans. In the human body, aging cells accumulate at diverse rates across multiple tissues [48]. This could also elucidate why, in subgroup analyses, individuals aged over 60 years demonstrated a more rapid rate of cellular aging.

### 4.1 Limitations and strengths

The strengths of our research include relying on data from a substantial population survey (NHANES) and utilizing a stringent random sampling process, which ensured that our findings represented the entire population. and using a variety of measures to estimate biological aging and provide robust associations.

However, several limitations of our study need to be considered. First, our analysis was conducted on cross-sectional data, lacking longitudinal repeated measures assessment of biological aging, thereby introducing the potential for reverse causality; hence, establishing causality is not possible. Second, serum cobalamin, MMA, and Hcy levels were ascertained from a single measurement, which may not accurately reflect the long-term status of the participants. Third, serum cobalamin, MMA, and Hcy were measured and analyzed at different stages of NHANES using diverse analytical methods. Despite statistical harmonization, the results fall short of those obtained by employing consistent experimental methods simultaneously. Fourth, we could not distinguish between congenital and postnatal increases in serum cobalamin and MMA. However, the rather low prevalence of hereditary methylmalonic academia [49] and functional cobalamin deficiency [15] means that they do not greatly influence our conclusions. Fifth, dietary cobalamin intake and cobalamin supplement use were derived from self-reports and were consequently subject to recall bias. Last, while efforts have been made to adjust for confounding factors, it is important to note that there may still be unaccounted potential confounders, such as cobalamin levels possibly correlated with poorer health, and participants with preexisting conditions might require additional supplements. Therefore, additional studies are necessary to validate our findings.

## 5 Conclusions

Decreased cobalamin sensitivity but not cobalamin insufficiency might be associated with biological aging acceleration. Further studies would improve the understanding of the underlying mechanisms between decreased cobalamin sensitivity and biological aging acceleration.

## Authors contributions

FT and SW conceived and designed the study. FT, HQ, YL JG, and ZH organized all data. FT and SW analyzed and visualized the results. FT and SW contributed to the manuscript. Reviewed and edited the manuscript, YZ and SF take responsibility for the integrity and accuracy of this analysis. All authors reviewed and edited the manuscript.

## Sources of funding

SW was funded by the 10.13039/501100001809National Natural Science Foundation of China (82200396), Natural Science Foundation of Heilongjiang Province of China (YQ2022H006), New era Longjiang outstanding doctoral key project (LJYXL2022-013), Cultivation Project of Second Affiliated Hospital of Harbin Medical University (PYMS2023-3); YZ was funded by the Gout Etiology and Functional Food Research Innovation Team, the North Medicine and Functional Food Characteristic Subject Project in Heilongjiang Province (HLJTSXK-2022-03), Foundation of Heilongjiang Educational Committee (2023-KYYWF-0620), and Project of Jiamusi University (JMSUQP23027), National Fund Cultivation Program of Jiamusi University (JMSUGPZR2022-022), Scientific and Technological Innovation Team of Jiamusi University (cxtd202101).

## Conflict of interest

There are no conflicts of interest to declare.
