# Supplementary material for: Decreased cobalamin sensitivity and biological aging acceleration in the general population
Source: J Nutr Health Aging. 2024 May 20;28(7):100262. doi: 10.1016/j.jnha.2024.100262 (PMC12433826; doi:10.1016/j.jnha.2024.100262)
Supplement: Supplementary file 1 [file mmc1.docx]

**Supplemental figures and tables**

**Figure S1.** **The Pearson correlations between** **biological age and chronological age**


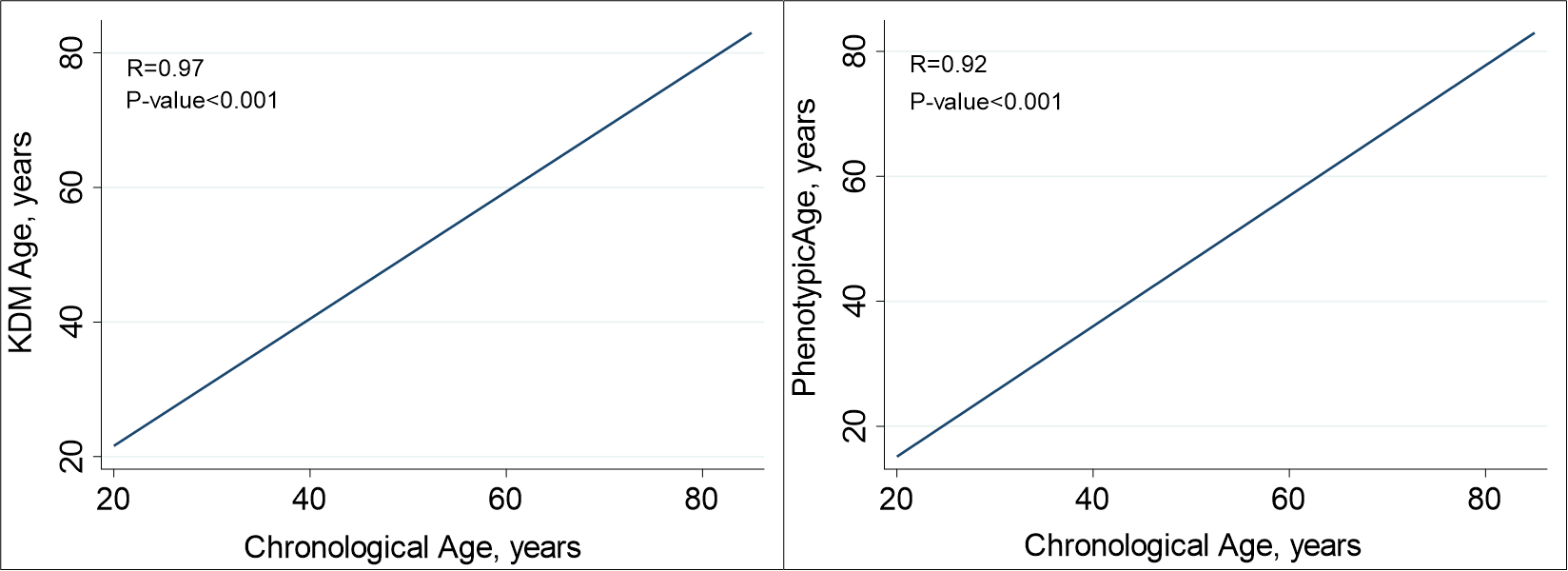


Abbreviations: KDM, Klemera-Doubal method;

1. The Pearson correlations between KDM Age and chronological age.
2. The Pearson correlations between Phenotypic Age and chronological age.

**Figure S2.** **The relationship between functional cobalamin deficiency and biological aging**


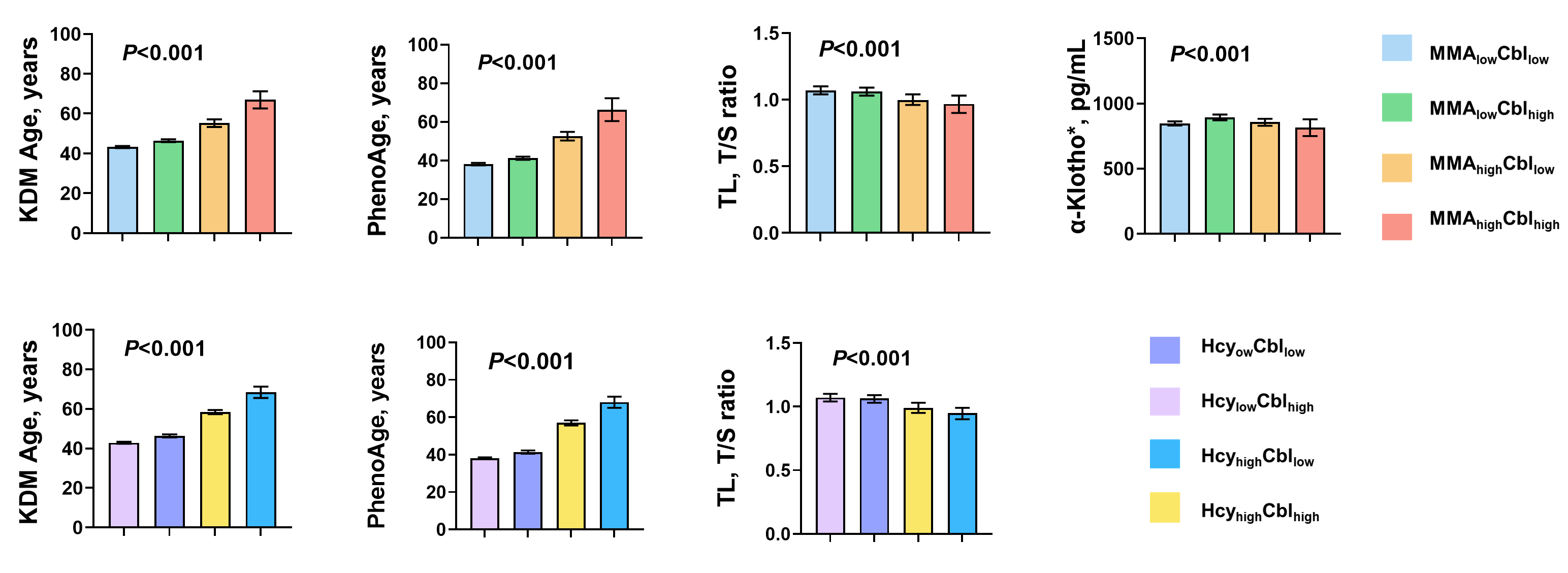


Abbreviations: KDM, Klemera-Doubal method; PhenoAge, Phenotypic Age; TL, Telomere Length; MMA, methylmalonic acid; Hcy, homocysteine; Cbl, cobalamin;

Data were adjusted for survey weights of NHANES;

The mean standard deviation of indicators related to functional cobalamin deficiency and biological aging; the p-values are calculated based on the chi-square test;

Error bars are 95%CIs;

*: Investigation of α-Klotho is conducted exclusively in males and females aged 40 to 79 years;

Combination of MMA or Hcy and cobalamin: Both indicators were categorized into high vs. low levels and combined into four groups according to the prespecified cutoff values (MMA >250 nmol/L or Hcy > 12.1μmol/l and cobalamin > 400 pg/mL).

**Table S1. Baseline characteristics of participants in NHANES 1999 – 2014**

| **Variables** | **1999-2000**  **Mean±SE or *n* (%)** | **2001-2002**  **Mean±SE or *n* (%)** | **2003-2004**  **Mean±SE or *n* (%)** | **2005-2006**  **Mean±SE or *n* (%)** | **2011-2012**  **Mean±SE or *n* (%)** | **2013-2014**  **Mean±SE or *n* (%)** |
| --- | --- | --- | --- | --- | --- | --- |
| Participants | 4116 | 4686 | 4442 | 4349 | 2454 | 2765 |
| Age, years | 44.9±0.4 | 45.4±0.5 | 46.4±0.5 | 46.7±0.7 | 56.1±0.3 | 56.4±0.2 |
| Sex, % |  |  |  |  |  |  |
| Male | 1925 (48.0) | 2233 (48.0) | 2153 (48.5) | 2083 (48.0) | 1195 (47.6) | 1288 (47.6) |
| Race/ethnicity, % |  |  |  |  |  |  |
| Mexican American | 1123 (6.3) | 999(7.2) | 892(7.7) | 892 (8.0) | 241 (5.7) | 401 (7.3) |
| Other Hispanic | 265 (8.3) | 203 (5.9) | 138 (3.7) | 129 (3.2) | 279 (5.4) | 260 (4.5) |
| Non-Hispanic White | 1846 (70.8) | 2486 (72.3) | 2371 (72.5) | 2191 (72.5) | 915 (73.1) | 120 3(71.9) |
| Non-Hispanic Black | 758 (10.4) | 844 (10.2) | 851 (10.8) | 966 (11.1) | 644 (9.1) | 519 (9.2) |
| Other Race | 124 (4.2) | 154 (4.4) | 190 (5.3) | 171 (5.2) | 375 (6.7) | 38 2(7.0) |
| Education, % |  |  |  |  |  |  |
| Less than high school | 1590 (23.8) | 1407 (19.3) | 1302 (18.1) | 1202 (17.4) | 625 (16.6) | 651 (14.8) |
| Highschool | 921 (25.8) | 1096 (25.2) | 1118 (27.0) | 1028 (24.7) | 522 (21.0) | 588 (20.9) |
| More than high school | 1605 (50.4) | 2183 (55.4) | 2022 (54.9) | 2119 (57.9) | 1297 (62.4) | 1526 (64.3) |
| Marital status, % |  |  |  |  |  |  |
| Married/Living with a partner | 2552 (63.1) | 2720 (59.1) | 2416 (57.9) | 2396 (58.5) | 1390 (62.2) | 1690 (66.7) |
| Widowed/Divorced/Separated | 746 (20.9) | 998 (23.4) | 1003 (23.3) | 1025 (22.8) | 377 (14.6) | 347 (10.7) |
| Never Married | 818 (16.0) | 968 (17.5) | 1023 (18.8) | 928 (18.7) | 687 (23.2) | 728 (22.6) |
| Poverty income ratio, % |  |  |  |  |  |  |
| ≤1.30 | 1638 (31.2) | 1481 (26.0) | 1472 (24.8) | 1296 (19.9) | 914 (23.0) | 1006 (24.0) |
| 1.31-3.5 | 1357 (31.9) | 1684 (33.0) | 1666 (35.9) | 1613 (36.1) | 757 (31.5) | 866 (30.4) |
| >3.5 | 1121 (36.9) | 1521 (41.0) | 1304 (39.3) | 1440 (44.0) | 783 (45.5) | 893 (45.6) |
| Body mass index, kg/m^2^ |  |  |  |  |  |  |
| >25 | 1299 (35.8) | 1426 (33.2) | 1367 (32.8) | 1300 (32.5) | 610 (24.1) | 693 (23.4) |
| 25 – 30 | 1438 (33.5) | 1660 (34.0) | 1548 (34.1) | 1473 (32.6) | 836 (36.2) | 928 (34.4) |
| ≥30 | 1379 (30.7) | 1600 (32.8) | 1527 (33.1) | 1576 (34.9) | 1008 (39.7) | 1144 (42.2) |
| Smoking status, % |  |  |  |  |  |  |
| Never | 2180 (50.9) | 2394 (50.4) | 2228 (49.3) | 2291(51.2) | 1308(51.6) | 1483(53.8) |
| Former | 1097 (24.8) | 1241 (24.8) | 1216 (25.4) | 1104(24.9) | 688(29.4) | 776(28.8) |
| Current | 839 (24.3) | 1051(24.8) | 998(25.3) | 954(23.9) | 458(19.0) | 506(17.4) |
| Physical activity, % |  |  |  |  |  |  |
| Less | 2095 (40.7) | 2011 (34.9) | 1869 (34.9) | 1704 (32.7) | 1357 (49.3) | 1460 (50.0) |
| Moderate | 948 (24.4) | 1256 (28.4) | 1460 (34.1) | 1331 (31.2) | 732 (32.7) | 853 (31.6) |
| Vigorous | 1073 (34.9) | 1419 (36.7) | 1113 (31.0) | 1314 (36.1) | 365 (18.0) | 452 (18.4) |
| Heavy drinking, % | 257 (8.5) | 299 (7.5) | 259 (6.8) | 300 (8.9) | 177 (10.3) | 164 (7.6) |
| Serum cobalamin, pg/ml | 407.3± 24.6 | 386.9± 10.4 | 388.9± 9.9 | 404.5± 10.7 | 410.2± 11.3 | 425.9± 8.4 |
| Cobalamin intake from foods, ug/day | 4.9±0.2 | 5.5±0.1 | 5.3±0.2 | 5.6±0.1 | 5.1±0.3 | 4.6±0.1 |
| Cobalamin supplements, % | 1469 (38.7) | 1800 (39.6) | 1680 (39.1) | - | 831 (36.8) | 987 (41.2) |
| Methylmalonic acid, nmol/L | 156.8±3.1 | 153.3±2.9 | 162.6±4.0 | - | 173.5±2.7 | 184.1±4.9 |
| Homocysteine, μmol/l | 8.1±0.1 | 8.7±0.1 | 9.1±0.1 | 8.4±0.1 | - | - |
| Telomere length, T/S ratio | 1.0±0.1 | 1.1±0.1 | - | - | - | - |
| α-Klotho^*^, pg/mL | - | - | - | - | 883.3±11.0 | 842.4±9.2 |
| KDM Age, years | 43.6±0.3 | 46.1±0.5 | 46.3±0.5 | 46.8±0.7 | - | - |
| Phenotypic Age, years | 38.4±0.4 | 40.8±0.5 | 42.1±0.5 | 43.4±0.8 | - | - |
| Type2 Diabetes, % | 463 (6.9) | 496 (7.4) | 526 (8.5) | 507 (8.6) | 518 (14.8) | 564 (16.0) |
| Cardiovascular diseases, % | 430 (7.8) | 493 (7.8) | 586 (9.6) | 481 (8.6) | 305 (10.4) | 341 (11.0) |
| Hypertension, % | 1674 (32.4) | 1825 (33.2) | 1902 (36.9) | 1691 (36.3) | 1320 (48.0) | 1487 (50.7) |

Percentages, means, and standard errors are adjusted for NHANES sampling weights. The observed numbers for categorical variables were unweighted;

Abbreviations: SE, standard errors; Heavy drinking (male ≥20g/day, female ≥10g/day); KDM, Klemera-Doubal method;

*Investigation of α-Klotho is conducted exclusively in males and females aged 40 to 79 years

**Table S2. Correlation matrix of cobalamin-related biomarkers and calculated blood indicators of biological aging in serum cobalamin less than 339 pg/mL**

| **Cbl <339 pg/mL** | MMA | Hcy | Deit Cbl | CRP | Scr | HbA1c | Alb | TC | SUN | ALP | SBP | L% | MCV | RDW | ALP | WBC | Age |
| --- | --- | --- | --- | --- | --- | --- | --- | --- | --- | --- | --- | --- | --- | --- | --- | --- | --- |
| MMA | 1.000 | - | - | - | - | - | - | - | - | - | - | - | - | - | - | - | - |
| Hcy | 0.492 | 1.000 |  | - | - | - | - | - | - | - | - | - | - | - | - | - | - |
| Deit Cbl | -0.008 | -0.017 | 1.000 | - | - | - | - | - | - | - | - | - | - | - | - | - | - |
| CRP | 0.006 | 0.010 | -0.018 | 1.000 | - | - | - | - | - | - | - | - | - | - | - | - | - |
| Scr | 0.148 | 0.369 | 0.015 | 0.019 | 1.000 | - | - | - | - | - | - | - | - | - | - | - | - |
| HbA1c | 0.080 | 0.102 | -0.027 | 0.106 | 0.064 | 1.000 | - | - | - | - | - | - | - | - | - | - | - |
| Alb | -0.027 | 0.075 | 0.016 | -0.274 | 0.001 | -0.088 | 1.000 | - | - | - | - | - | - | - | - | - | - |
| TC | 0.004 | -0.002 | -0.005 | -0.006 | -0.045 | 0.064 | -0.043 | 1.000 | - | - | - | - | - | - | - | - | - |
| SUN | 0.143 | 0.363 | 0.024 | 0.011 | 0.515 | 0.169 | 0.080 | -0.022 | 1.000 | - | - | - | - | - | - | - | - |
| ALP | 0.041 | 0.068 | -0.019 | 0.183 | 0.013 | 0.127 | -0.132 | 0.103 | 0.056 | 1.000 | - | - | - | - | - | - | - |
| SBP | 0.128 | 0.108 | -0.011 | 0.099 | 0.077 | 0.792 | -0.071 | 0.041 | 0.160 | 0.121 | 1.000 | - | - | - | - | - | - |
| L% | -0.056 | -0.040 | -0.022 | -0.164 | -0.042 | 0.048 | 0.144 | 0.004 | -0.081 | -0.072 | -0.017 | 1.000 | - | - | - | - | - |
| MCV | 0.069 | 0.141 | 0.022 | -0.082 | 0.071 | -0.108 | 0.085 | 0.034 | 0.088 | -0.026 | -0.026 | -0.034 | 1.000 | - | - | - | - |
| RDW | 0.080 | 0.190 | -0.019 | 0.166 | 0.159 | 0.102 | -0.239 | -0.045 | 0.096 | 0.114 | 0.064 | -0.055 | -0.371 | 1.000 | - | - | - |
| ALP | 0.033 | 0.060 | -0.012 | 0.196 | 0.003 | 0.122 | -0.125 | 0.114 | 0.061 | 1.000 | 0.116 | -0.076 | -0.026 | 0.105 | 1.000 | - | - |
| WBC | -0.003 | -0.044 | -0.008 | 0.194 | -0.040 | 0.041 | -0.125 | 0.066 | -0.048 | 0.094 | 0.011 | -0.244 | -0.021 | 0.041 | 0.085 | 1.000 | - |
| Age | 0.124 | 0.372 | -0.031 | 0.052 | 0.195 | 0.277 | -0.068 | 0.098 | 0.426 | 0.086 | 0.227 | -0.061 | 0.194 | 0.184 | 0.082 | -0.097 | 1.000 |

Correlation analysis was conducted using the Pearson method.

Abbreviations: Cbl, cobalamin; MMA, Methylmalonic acid; Hcy, Homocysteine; Diet Cbl, Cobalamin intake from foods; CRP, C-reactive protein; SCr, serum creatinine; HbA1c, glycosylated hemoglobin; Alb, Serum albumin; TC, Serum total cholesterol; SUN, Serum urea nitrogen; ALP, Serum alkaline phosphatase; SBP, Systolic blood pressure; L%, Lymphocyte percent; MCV, Mean cell volume; RDW, Red cell distribution width; ALP, Alkaline phosphatase; WBC, White blood cell count;

**Table S3. Correlation matrix of cobalamin-related biomarkers and calculated blood indicators of biological aging in serum cobalamin more than 339 pg/mL**

| **Cbl ≥339 pg/mL** | MMA | Hcy | Deit Cbl | CRP | Scr | HbA1c | Alb | TC | SUN | ALP | SBP | L% | MCV | RDW | ALP | WBC | Age |
| --- | --- | --- | --- | --- | --- | --- | --- | --- | --- | --- | --- | --- | --- | --- | --- | --- | --- |
| MMA | 1.000 | - | - | - | - | - | - | - | - | - | - | - | - | - | - | - | - |
| Hcy | 0.492 | 1.000 | - | - | - | - | - | - | - | - | - | - | - | - | - | - | - |
| Deit Cbl | -0.006 | -0.017 | 1.000 | - | - | - | - | - | - | - | - | - | - | - | - | - | - |
| CRP | 0.006 | 0.010 | -0.018 | 1.000 | - | - | - | - | - | - | - | - | - | - |  | - | - |
| Scr | 0.143 | 0.369 | 0.014 | 0.019 | 1.000 | - | - | - | - | - | - | - | - | - | - | - | - |
| HbA1c | 0.066 | 0.102 | -0.025 | 0.106 | 0.072 | 1.000 | - | - | - | - | - | - | - | - | - | - | - |
| Alb | -0.025 | 0.075 | 0.017 | -0.274 | -0.016 | -0.091 | 1.000 | - | - | - | - | - | - | - | - | - | - |
| TC | 0.005 | -0.002 | -0.005 | -0.006 | -0.068 | 0.052 | -0.026 | 1.000 | - | - | - | - | - | - | - | - | - |
| SUN | 0.138 | 0.363 | 0.024 | 0.011 | 0.520 | 0.168 | 0.062 | -0.034 | 1.000 | - | - | - | - | - | - | - | - |
| ALP | 0.046 | 0.068 | -0.022 | 0.183 | 0.024 | 0.130 | -0.141 | 0.097 | 0.053 | 1.000 | - | - | - | - | - | - | - |
| SBP | 0.106 | 0.108 | -0.008 | 0.099 | 0.087 | 0.800 | -0.077 | 0.030 | 0.158 | 0.128 | 1.000 | - | - | - | - | - | - |
| L% | -0.064 | -0.040 | -0.020 | -0.164 | -0.052 | 0.048 | 0.134 | 0.005 | -0.085 | -0.072 | -0.019 | 1.000 | - | - | - | - | - |
| MCV | 0.069 | 0.141 | 0.020 | -0.082 | 0.062 | -0.115 | 0.085 | 0.028 | 0.078 | -0.022 | -0.037 | -0.038 | 1.000 | - | - | - | - |
| RDW | 0.065 | 0.190 | -0.019 | 0.166 | 0.165 | 0.104 | -0.232 | -0.054 | 0.104 | 0.104 | 0.062 | -0.054 | -0.386 | 1.000 | - | - | - |
| ALP | 0.040 | 0.060 | -0.016 | 0.196 | 0.017 | 0.125 | -0.137 | 0.106 | 0.056 | 1.000 | 0.125 | -0.076 | -0.020 | 0.093 | 1.000 |  | - |
| WBC | 0.001 | -0.044 | -0.008 | 0.194 | -0.039 | 0.042 | -0.123 | 0.064 | -0.043 | 0.107 | 0.016 | -0.264 | -0.022 | 0.034 | 0.103 | 1.000 | - |
| Age | 0.113 | 0.372 | -0.030 | 0.052 | 0.193 | 0.265 | -0.065 | 0.083 | 0.415 | 0.079 | 0.214 | -0.059 | 0.183 | 0.190 | 0.072 | -0.107 | 1.000 |

Correlation analysis was conducted using the Pearson method.

Abbreviations: Cbl, cobalamin; MMA, Methylmalonic acid; Hcy, Homocysteine; Diet Cbl, Cobalamin intake from foods; CRP, C-reactive protein; SCr, serum creatinine; HbA1c, glycosylated hemoglobin; Alb, Serum albumin; TC, Serum total cholesterol; SUN, Serum urea nitrogen; ALP, Serum alkaline phosphatase; SBP, Systolic blood pressure; L%, Lymphocyte percent; MCV, Mean cell volume; RDW, Red cell distribution width; ALP, Alkaline phosphatase; WBC, White blood cell count;

**Table S4. The relationship between cobalamin or its intake or its supplements and biological aging**

|  | **Crude** | | **Model 1** | | **Model 2** | |
| --- | --- | --- | --- | --- | --- | --- |
|  | **β 95%CI** | ***P*** | **β 95%CI** | ***P*** | **β 95%CI** | ***P*** |
| **Serum cobalamin, pg/ml** | |  |  |  |  |  |
| KDMAge | 1.85 (1.39 to 2.32) | <0.001 | 0.34 (0.16 to 0.52) | <0.001 | 0.42 (0.25 to 0.58) | <0.001 |
| Phenotypic Age | 1.47 (0.89 to 2.05) | <0.001 | -0.29 (-0.52 to -0.06) | 0.01 | 0.06 (-0.13 to 0.25) | 0.56 |
| KDMAccel | 0.30 (0.11 to 0.48) | <0.001 | 0.34 (0.16 to 0.52) | <0.001 | 0.42 (0.25 to 0.58) | <0.001 |
| PhenoAgeAccel | -0.07 (-0.30 to 0.15) | 0.52 | -0.29 (-0.52 to -0.06) | 0.01 | 0.06 (-0.13 to 0.25) | 0.56 |
| Telomere length | -0.004 (-0.015 to 0.006) | 0.397 | 0.006 (-0.005 to 0.017) | 0.293 | 0.003 (-0.008 to 0.014) | 0.553 |
| α-Klotho | 27.77 (16.71 to 38.84) | <0.001 | 27.55 (16.60 to 38.51) | <0.001 | 23.18 (12.56 to 33.80) | <0.001 |
| PhenoAge advancement* | 1.00 (0.95 to 1.07) | 0.87 | 1.04 (0.98 to 1.10) | 0.19 | 1.08 (1.02 to 1.15) | 0.01 |
| **Cobalamin intake from food, ug/day** | |  |  |  |  |  |
| KDMAge | -0.23 (-0.47 to 0.00) | 0.05 | 0.23 (0.16 to 0.29) | <0.001 | 0.24 (0.17 to 0.30) | <0.001 |
| Phenotypic Age | -0.45 (-0.70 to -0.19) | <0.001 | 0.08 (-0.02 to 0.17) | 0.13 | 0.15 (0.07 to 0.23) | <0.001 |
| KDMAccel | 0.31 (0.25 to 0.37) | <0.001 | 0.23 (0.16 to 0.29) | <0.001 | 0.24 (0.17 to 0.30) | <0.001 |
| PhenoAgeAccel | 0.10 (0.01 to 0.18) | 0.03 | 0.08 (-0.02 to 0.17) | 0.13 | 0.15 (0.07 to 0.23) | <0.001 |
| Telomere length | 0.006 (0.005 to 0.011) | 0.043 | 0.010 (0.001 to 0.011) | 0.061 | 0.005 (-0.001 to 0.010) | 0.086 |
| α-Klotho | 3.58 (-7.52 to 14.69) | 0.52 | 7.76 (-3.65 to 19.18) | 0.18 | 5.73 (-4.39 to 15.86) | 0.26 |
| PhenoAge advancement* | 1.15 (1.12 to 1.18) | <0.001 | 1.11 (1.07 to 1.14) | <0.001 | 1.11 (1.08 to 1.15) | <0.001 |
| **Cobalamin supplements, %** | |  |  |  |  |  |
| KDMAge | 4.01 (3.23 to 4.78) | <0.001 | -0.23 (-0.46 to -0.01) | 0.04 | -0.04 (-0.28 to 0.20) | 0.72 |
| Phenotypic Age | 3.76 (2.89 to 4.63) | <0.001 | -0.83 (-1.19 to -0.47) | <0.001 | 0.02 (-0.35 to 0.39) | 0.91 |
| KDMAccel | -0.63 (-0.86 to -0.40) | <0.001 | -0.23 (-0.46 to -0.01) | 0.04 | -0.04 (-0.28 to 0.20) | 0.72 |
| PhenoAgeAccel | -0.91 (-1.26 to -0.56) | <0.001 | -0.83 (-1.19 to -0.47) | <0.001 | 0.02 (-0.35 to 0.39) | 0.91 |
| Telomere length | -0.028 (-0.045 to -0.011) | 0.002 | -0.001 (-0.018 to 0.017) | 0.936 | -0.007 (-0.023 to 0.008) | 0.352 |
| α-Klotho | 0.15 (-16.85 to 17.14) | 0.99 | 8.30 (-10.40 to 27.00) | 0.37 | -0.73 (-17.68 to 16.22) | 0.93 |
| PhenoAge advancement* | 0.82 (0.76 to 0.88) | <0.001 | 0.95 (0.88 to 1.04) | 0.26 | 1.01 (0.91 to 1.12) | 0.88 |

Abbreviations: KDM, Klemera-Doubal method; KDMAccel, KDM Age acceleration; PhenoAgeAccel, Phenotypic Age acceleration; PhenoAge advancement, Phenotypic Age advancement;

Model 1: age (continuous), sex (female, male), race/ ethnicity (Mexican-American, other Hispanic, non-Hispanic white, non-Hispanic black, another race),

Model 2: age (continuous), sex (female, male), race/ ethnicity (Mexican-American, other Hispanic, non-Hispanic white, non-Hispanic black, other race), education level (less than high school, high school, more than high school), marital status (married/cohabitating, divorced/widowed/separated, never married), Poverty income ratio (<1.3, 1.3-3.5, >3.5). body mass index (<25.0 kg/m²), 25.0-29.9 kg/m², ≥30.0 kg/m²), smoking status (never, former, current), heavy alcohol consumption (male ≥20g/day, female ≥10g/day), physical activity (less, moderate, vigorous), Type 2 Diabetes (Yes/no), hypertension (Yes/no) and cardiovascular diseases (Yes/no).

*: PhenoAge advancement is a binary variable, the results are shown as OR, 95%CI

Investigation of α-Klotho is conducted exclusively in males and females aged 40 to 79 years

**Table S5. The relationship between methylmalonic acid or homocysteine and biological aging**

|  | **Crude** | | **Model 1** | | **Model 2** | |
| --- | --- | --- | --- | --- | --- | --- |
|  | **β 95%CI** | ***P*** | **β 95%CI** | ***P*** | **β 95%CI** | ***P*** |
| **Methylmalonic acid, nmol/L** | |  |  |  |  |  |
| KDMAge | 8.11 (7.46 to 8.77) | <0.001 | 1.21 (0.86 to 1.56) | <0.001 | 1.12 (0.80 to 1.45) | <0.001 |
| Phenotypic Age | 9.58 (8.74 to 10.42) | <0.001 | 2.19 (1.54 to 2.84) | <0.001 | 1.86 (1.25 to 2.47) | <0.001 |
| KDMAccel | 0.68 (0.36 to 1.00) | <0.001 | 1.21 (0.86 to 1.56) | <0.001 | 1.12 (0.80 to 1.45) | <0.001 |
| PhenoAgeAccel | 2.14 (1.55 to 2.73) | <0.001 | 2.19 (1.54 to 2.84) | <0.001 | 1.86 (1.25 to 2.47) | <0.001 |
| Telomere length | -0.048 (-0.063 to -0.034) | <0.001 | -0.004 (-0.018 to 0.01) | 0.557 | -0.003 (-0.017 to 0.011) | 0.653 |
| α-Klotho | -18.37 (-32.55 to -4.20) | 0.01 | -5.99 (-18.90 to 6.91) | 0.35 | -4.02 (-16.29 to 8.25) | 0.51 |
| PhenoAge advancement* | 1.02 (0.94 to 1.10) | 0.65 | 1.23 (1.14 to 1.33) | <0.001 | 1.24 (1.14 to 1.35) | <0.001 |
| **Homocysteine, μmol/l** | |  |  |  |  |  |
| KDM Age | 14.22 (13.50 to 14.94) | <0.001 | 1.39 (1.08 to 1.69) | <0.001 | 1.45 (1.18 to 1.73) | <0.001 |
| Phenotypic Age | 16.67 (15.79 to 17.56) | <0.001 | 2.69 (2.18 to 3.20) | <0.001 | 2.07 (1.63 to 2.51) | <0.001 |
| KDMAccel | 0.60 (0.36 to 0.84) | <0.001 | 1.39 (1.08 to 1.69) | <0.001 | 1.45 (1.18 to 1.73) | <0.001 |
| PhenoAgeAccel | 3.04 (2.65 to 3.43) | <0.001 | 2.69 (2.18 to 3.20) | <0.001 | 2.07 (1.63 to 2.51) | <0.001 |
| Telomere length | -0.078 (-0.102 to -0.055) | <0.001 | 0.01 (-0.017 to 0.028) | 0.645 | 0.01 (-0.01 to 0.03) | 0.50 |
| PhenoAge advancement* | 0.95 (0.89 to 1.01) | 0.09 | 1.26 (1.16 to 1.36) | <0.001 | 1.32 (1.21 to 1.44) | <0.001 |

Abbreviations: KDM, Klemera-Doubal method; KDMAccel, KDM Age acceleration; PhenoAgeAccel, Phenotypic Age acceleration; PhenoAge advancement, Phenotypic Age advancement;

Model 1: age (continuous), sex (female, male), race/ ethnicity (Mexican-American, other Hispanic, non-Hispanic white, non-Hispanic black, another race),

Model 2: age (continuous), sex (female, male), race/ ethnicity (Mexican-American, other Hispanic, non-Hispanic white, non-Hispanic black, other race), education level (less than high school, high school, more than high school), marital status (married/cohabitating, divorced/widowed/separated, never married), Poverty income ratio (<1.3, 1.3-3.5, >3.5). body mass index (<25.0 kg/m²), 25.0-29.9 kg/m², ≥30.0 kg/m²), smoking status (never, former, current), heavy alcohol consumption (male ≥20g/day, female ≥10g/day), physical activity (less, moderate, vigorous), Type 2 Diabetes (Yes/no), hypertension (Yes/no) and cardiovascular diseases (Yes/no);

*: PhenoAge advancement is a binary variable, the results are shown as OR, 95%CI;

Investigation of α-Klotho is conducted exclusively in males and females aged 40 to 79 years.

**Table S6. The relationship between functional cobalamin deficiency based on methylmalonic acid and cobalamin and biological aging**

|  | **MMA_low_Cbl_low_ (n=10,266)** | **MMA_low_Cbl_high_ (n=6,111)** | | **MMA_high_Cbl_low_ (n=1,544)** | | **MMA_high_Cbl_high_ (n=384)** | |
| --- | --- | --- | --- | --- | --- | --- | --- |
|  | **β 95%CI** | **β 95%CI** | ***P*** | **β 95%CI** | ***P*** | **β 95%CI** | ***P*** |
| **KDM Age** |  |  |  |  |  |  |  |
| Crude | Ref. | 3.23 (2.53 to 3.92) | P<0.001 | 11.98 (9.99 to 13.96) | P<0.001 | 23.71 (19.45 to 27.97) | P<0.001 |
| Model 1 | Ref. | 0.16 (-0.08 to 0.41) | 0.19 | 0.96 (0.51 to 1.41) | P<0.001 | 8.47 (6.23 to 10.72) | P<0.001 |
| Model 2 | Ref. | 0.27 (0.03 to 0.51) | 0.03 | 0.85 (0.41 to 1.29) | P<0.001 | 7.97 (5.77 to 10.17) | P<0.001 |
| **Phenotypic Age** |  |  |  |  |  |  |  |
| Crude | Ref. | 3.01 (2.24 to 3.78) | P<0.001 | 14.46 (12.18 to 16.74) | P<0.001 | 28.19 (22.32 to 34.06) | P<0.001 |
| Model 1 | Ref. | -0.44 (-0.70 to -0.19) | P<0.001 | 2.47 (1.83 to 3.10) | P<0.001 | 11.68 (7.36 to 16.00) | P<0.001 |
| Model 2 | Ref. | -0.05 (-0.30 to 0.20) | 0.68 | 1.87 (1.24 to 2.50) | P<0.001 | 10.68 (6.58 to 14.79) | P<0.001 |
| **KDMAccel** |  |  |  |  |  |  |  |
| Crude | Ref. | 0.01 (-0.25 to 0.27) | 0.91 | 0.24 (-0.19 to 0.67) | 0.27 | 7.47 (5.24 to 9.70) | P<0.001 |
| Model 1 | Ref. | 0.16 (-0.08 to 0.41) | 0.19 | 0.96 (0.51 to 1.41) | P<0.001 | 8.47 (6.23 to 10.72) | P<0.001 |
| Model 2 | Ref. | 0.27 (0.03 to 0.51) | 0.03 | 0.85 (0.41 to 1.29) | P<0.001 | 7.97 (5.77 to 10.17) | P<0.001 |
| **PhenoAgeAccel** |  |  |  |  |  |  |  |
| Crude | Ref. | -0.18 (-0.45 to 0.09) | 0.18 | 2.75 (2.14 to 3.36) | P<0.001 | 12.13 (7.81 to 16.44) | P<0.001 |
| Model 1 | Ref. | -0.44 (-0.70 to -0.19) | P<0.001 | 2.47 (1.83 to 3.10) | P<0.001 | 11.68 (7.36 to 16.00) | P<0.001 |
| Model 2 | Ref. | -0.05 (-0.30 to 0.20) | 0.68 | 1.87 (1.24 to 2.50) | P<0.001 | 10.68 (6.58 to 14.79) | P<0.001 |
| **Telomere length** |  |  |  |  |  |  |  |
| Crude | Ref. | -0.008 (-0.025 to 0.008) | 0.309 | -0.071 (-0.096 to -0.046) | P<0.001 | -0.101 (-0.162 to -0.04) | 0.002 |
| Model 1 | Ref. | 0.007 (-0.009 to 0.022) | 0.381 | -0.005 (-0.036 to 0.026) | 0.752 | -0.019 (-0.072 to 0.035) | 0.485 |
| Model 2 | Ref. | 0.003 (-0.012 to 0.019) | 0.659 | -0.003 (-0.034 to 0.028) | 0.839 | -0.020 (-0.079 to 0.039) | 0.488 |
| **α-Klotho** |  |  |  |  |  |  |  |
| Crude | Ref. | 45.59 (28.16 to 63.03) | P<0.001 | 8.32 (-24.00 to 40.64) | 0.60 | -32.89 (-99.65 to 33.87) | 0.32 |
| Model 1 | Ref. | 45.77 (27.10 to 64.43) | P<0.001 | 22.49 (-8.47 to 53.45) | 0.15 | -13.28 (-78.81 to 52.25) | 0.68 |
| Model 2 | Ref. | 37.16 (19.03 to 55.29) | P<0.001 | 26.03 (-3.39 to 55.46) | 0.08 | -23.43 (-88.54 to 41.69) | 0.47 |
| **PhenoAge advancement*** | |  |  |  |  |  |  |
| Crude | 1.00 (Ref.) | 0.91 (0.81 to 1.04) | 0.15 | 0.87 (0.74 to 1.02) | 0.09 | 1.70 (1.22 to 2.37) | P<0.001 |
| Model 1 | 1.00 (Ref.) | 0.98 (0.86 to 1.11) | 0.76 | 1.16 (0.97 to 1.38) | 0.11 | 2.71 (1.89 to 3.88) | P<0.001 |
| Model 2 | 1.00 (Ref.) | 1.04 (0.90 to 1.19) | 0.61 | 1.17 (0.96 to 1.42) | 0.13 | 2.79 (1.82 to 4.29) | P<0.001 |

Abbreviations: MMA, methylmalonic acid; Cbl, cobalamin; KDM, Klemera-Doubal method; KDMAccel, KDM Age acceleration; PhenoAgeAccel, Phenotypic Age acceleration; PhenoAge advancement, Phenotypic Age advancement;

Model 1: age (continuous), sex (female, male), race/ ethnicity (Mexican-American, other Hispanic, non-Hispanic white, non-Hispanic black, another race),

Model 2: age (continuous), sex (female, male), race/ ethnicity (Mexican-American, other Hispanic, non-Hispanic white, non-Hispanic black, other race), education level (less than high school, high school, more than high school), marital status (married/cohabitating, divorced/widowed/separated, never married), Poverty income ratio (<1.3, 1.3-3.5, >3.5). body mass index (<25.0 kg/m²), 25.0-29.9 kg/m², ≥30.0 kg/m²), smoking status (never, former, current), heavy alcohol consumption (male ≥20g/day, female ≥10g/day), physical activity (less, moderate, vigorous), Type 2 Diabetes (Yes/no), hypertension (Yes/no) and cardiovascular diseases (Yes/no).

*: PhenoAge advancement is a binary variable, the results are shown as OR, 95%CI;

Investigation of α-Klotho is conducted exclusively in males and females aged 40 to 79 years;

Combination of MMA and cobalamin: The indicator was categorized into high vs. low levels and combined into four groups according to the prespecified cutoff values (MMA >250 nmol/L and cobalamin >400 pg/mL).

**Table S7. The relationship between functional cobalamin deficiency based on homocysteine and cobalamin and biological aging**

|  | **Hcy_low_Cbl_low_ (n=9,594)** | **Hcy_low_Cbl_high_(n=5,732)** | | **Hcy_high_Cbl_low_ (n=1,700)** | | **Hcy_high_Cbl_high_ (n=532)** | |
| --- | --- | --- | --- | --- | --- | --- | --- |
|  | **β 95%CI** | **β 95%CI** | ***P*** | **β 95%CI** | ***P*** | **β 95%CI** | ***P*** |
| **KDM Age** |  |  |  |  |  |  |  |
| Crude | Ref. | 3.48 (2.90 to 4.06) | P<0.001 | 15.54 (14.36 to 16.73) | P<0.001 | 25.55 (22.70 to 28.40) | P<0.001 |
| Model 1 | Ref. | 0.18 (-0.01 to 0.37) | 0.07 | 1.20 (0.81 to 1.59) | P<0.001 | 7.45 (5.97 to 8.93) | P<0.001 |
| Model 2 | Ref. | 0.30 (0.11 to 0.49) | P<0.001 | 1.24 (0.86 to 1.62) | P<0.001 | 6.99 (5.71 to 8.27) | P<0.001 |
| **Phenotypic Age** |  |  |  |  |  |  |  |
| Crude | Ref. | 3.29 (2.60 to 3.98) | P<0.001 | 18.95 (17.49 to 20.41) | P<0.001 | 29.97 (26.96 to 32.97) | P<0.001 |
| Model 1 | Ref. | -0.41 (-0.67 to -0.16) | P<0.001 | 3.18 (2.61 to 3.75) | P<0.001 | 9.43 (7.87 to 10.99) | P<0.001 |
| Model 2 | Ref. | 0.01 (-0.23 to 0.24) | 0.94 | 2.58 (2.11 to 3.05) | P<0.001 | 8.07 (6.62 to 9.52) | P<0.001 |
| **KDMAccel** |  |  |  |  |  |  |  |
| Crude | Ref. | -0.01 (-0.22 to 0.19) | 0.90 | 0.33 (-0.01 to 0.67) | 0.05 | 6.37 (4.92 to 7.81) | P<0.001 |
| Model 1 | Ref. | 0.18 (-0.01 to 0.37) | 0.07 | 1.20 (0.81 to 1.59) | P<0.001 | 7.45 (5.97 to 8.93) | P<0.001 |
| Model 2 | Ref. | 0.30 (0.11 to 0.49) | P<0.001 | 1.24 (0.86 to 1.62) | P<0.001 | 6.99 (5.71 to 8.27) | P<0.001 |
| **PhenoAgeAccel** |  |  |  |  |  |  |  |
| Crude | Ref. | -0.17 (-0.43 to 0.09) | 0.19 | 3.78 (3.26 to 4.31) | P<0.001 | 10.41 (8.81 to 12.01) | P<0.001 |
| Model 1 | Ref. | -0.41 (-0.67 to -0.16) | P<0.001 | 3.18 (2.61 to 3.75) | P<0.001 | 9.43 (7.87 to 10.99) | P<0.001 |
| Model 2 | Ref. | 0.01 (-0.23 to 0.24) | 0.94 | 2.58 (2.11 to 3.05) | P<0.001 | 8.07 (6.62 to 9.52) | P<0.001 |
| **Telomere length** |  |  |  |  |  |  |  |
| Crude | Ref. | -0.008 (-0.028 to 0.012) | 0.437 | -0.08 (-0.117 to -0.043) | P<0.001 | -0.123 (-0.16 to -0.086) | P<0.001 |
| Model 1 | Ref. | 0.009 (-0.010 to 0.028) | 0.336 | 0.011 (-0.024 to 0.046) | 0.535 | -0.021 (-0.062 to 0.020) | 0.301 |
| Model 2 | Ref. | 0.005 (-0.014 to 0.024) | 0.574 | 0.012 (-0.020 to 0.045) | 0.449 | -0.017 (-0.057 to 0.023) | 0.399 |
| **PhenoAge advancement*** |  |  |  |  |  |  |  |
| Crude | 1.00 (Ref.) | 0.91 (0.83 to 1.01) | 0.08 | 0.84 (0.75 to 0.94) | P<0.001 | 1.90 (1.47 to 2.45) | P<0.001 |
| Model 1 | 1.00 (Ref.) | 0.99 (0.90 to 1.10) | 0.90 | 1.19 (1.06 to 1.34) | 0.01 | 3.27 (2.45 to 4.38) | P<0.001 |
| Model 2 | 1.00 (Ref.) | 1.06 (0.95 to 1.18) | 0.32 | 1.27 (1.10 to 1.47) | P<0.001 | 3.68 (2.73 to 4.96) | P<0.001 |

Abbreviations: Hcy, homocysteine; Cbl, cobalamin; KDM, Klemera-Doubal method; KDMAccel, KDM Age acceleration; PhenoAgeAccel, Phenotypic Age acceleration; PhenoAge advancement, Phenotypic Age advancement;

Model 1: age (continuous), sex (female, male), race/ ethnicity (Mexican-American, other Hispanic, non-Hispanic white, non-Hispanic black, another race),

Model 2: age (continuous), sex (female, male), race/ ethnicity (Mexican-American, other Hispanic, non-Hispanic white, non-Hispanic black, other race), education level (less than high school, high school, more than high school), marital status (married/cohabitating, divorced/widowed/separated, never married), Poverty income ratio (<1.3, 1.3-3.5, >3.5). body mass index (<25.0 kg/m²), 25.0-29.9 kg/m², ≥30.0 kg/m²), smoking status (never, former, current), heavy alcohol consumption (male ≥20g/day, female ≥10g/day), physical activity (less, moderate, vigorous), Type 2 Diabetes (Yes/no), hypertension (Yes/no) and cardiovascular diseases (Yes/no).

*: PhenoAge advancement is a binary variable, the results are shown as OR, 95%CI

Investigation of α-Klotho is conducted exclusively in males and females aged 40 to 79 years

Combination of Hcy and cobalamin: The indicator was categorized into high vs. low levels and combined into four groups according to the prespecified cutoff values (Hcy>12.1μmol/l and cobalamin >400 pg/mL)

**Table S8.** **The relationship between** **the cobalamin insensitivity index based on** **methylmalonic acid and cobalamin and biological Aging**

|  | **Crude** | | **Model 1** | | **Model 2** | |
| --- | --- | --- | --- | --- | --- | --- |
|  | **β 95%CI** | ***P*** | **β 95%CI** | ***P*** | **β 95%CI** | ***P*** |
| **KDM Age** | 7.11 (6.60 to 7.62) | P<0.001 | 1.09 (0.82 to 1.36) | P<0.001 | 1.10 (0.84 to 1.35) | P<0.001 |
| **Phenotypic Age** | 7.96 (7.31 to 8.60) | P<0.001 | 1.38 (0.85 to 1.92) | P<0.001 | 1.39 (0.89 to 1.90) | P<0.001 |
| **KDMAccel** | 0.68 (0.43 to 0.94) | P<0.001 | 1.09 (0.82 to 1.36) | P<0.001 | 1.10 (0.84 to 1.35) | P<0.001 |
| **PhenoAgeAccel** | 1.54 (1.04 to 2.03) | P<0.001 | 1.38 (0.85 to 1.92) | P<0.001 | 1.39 (0.89 to 1.90) | P<0.001 |
| **Telomere length** | -0.037 (-0.046 to -0.027) | P<0.001 | 0.001 (-0.008 to 0.010) | 0.771 | 0.001 (-0.010 to 0.010) | 0.976 |
| **α-Klotho** | 13.00 (0.80 to 25.21) | 0.04 | 21.03 (8.43 to 33.63) | P<0.001 | 18.15 (6.24 to 30.06) | P<0.001 |
| **PhenoAge advancement*** | 1.00 (0.95 to 1.06) | 0.93 | 1.17 (1.10 to 1.25) | P<0.001 | 1.21 (1.13 to 1.30) | P<0.001 |

Abbreviations: MMA, methylmalonic acid; KDM, Klemera-Doubal method; KDMAccel, KDM Age acceleration; PhenoAgeAccel, Phenotypic Age acceleration; PhenoAge advancement, Phenotypic Age advancement;

Model 1: age (continuous), sex (female, male), race/ ethnicity (Mexican-American, other Hispanic, non-Hispanic white, non-Hispanic black, another race);

Model 2: age (continuous), sex (female, male), race/ ethnicity (Mexican-American, other Hispanic, non-Hispanic white, non-Hispanic black, other race), education level (less than high school, high school, more than high school), marital status (married/cohabitating, divorced/widowed/separated, never married), Poverty income ratio (<1.3, 1.3-3.5, >3.5). body mass index (<25.0 kg/m², 25.0-29.9 kg/m², ≥30.0 kg/m²), smoking status (never, former, current), heavy alcohol consumption (male ≥20g/day, female ≥10g/day), physical activity (less, moderate, vigorous), Type 2 Diabetes (Yes/no), hypertension (Yes/no) and cardiovascular diseases (Yes/no);

*: PhenoAge advancement is a binary variable, the results are shown as OR, 95%CI;

Investigation of α-Klotho is conducted exclusively in males and females aged 40 to 79 years;

Cobalamin insensitivity index estimated by methylmalonic acid and cobalamin;

Cobalamin insensitivity index based on methylmalonic acid and cobalamin: a multiplicative term by methylmalonic acid and cobalamin divided by 100.

**Table S9. The relationship between the cobalamin insensitivity index** **based on homocysteine and cobalamin and** **biological aging**

|  | **Crude** | | **Model 1** | | **Model 2** | |
| --- | --- | --- | --- | --- | --- | --- |
|  | **β 95%CI** | ***P*** | **β 95%CI** | ***P*** | **β 95%CI** | ***P*** |
| **KDM Age** | 8.15 (7.64 to 8.65) | P<0.001 | 0.91 (0.73 to 1.09) | P<0.001 | 0.99 (0.82 to 1.15) | P<0.001 |
| **Phenotypic Age** | 8.97 (8.37 to 9.57) | P<0.001 | 0.83 (0.60 to 1.06) | P<0.001 | 0.88 (0.68 to 1.08) | P<0.001 |
| **KDMAccel** | 0.53 (0.36 to 0.70) | P<0.001 | 0.91 (0.73 to 1.09) | P<0.001 | 0.99 (0.82 to 1.15) | P<0.001 |
| **PhenoAgeAccel** | 1.35 (1.13 to 1.57) | P<0.001 | 0.83 (0.60 to 1.06) | P<0.001 | 0.88 (0.68 to 1.08) | P<0.001 |
| **telomere length** | -0.04 (-0.054 to -0.027) | P<0.001 | 0.008 (-0.006 to 0.022) | 0.281 | 0.006 (-0.009 to 0.02) | 0.411 |
| **PhenoAge advancement*** | 0.98 (0.93 to 1.03) | 0.47 | 1.14 (1.08 to 1.21) | P<0.001 | 1.21 (1.14 to 1.29) | P<0.001 |

Abbreviations: Hcy, homocysteine; KDM, Klemera-Doubal method; KDMAccel, KDM Age acceleration; PhenoAgeAccel, Phenotypic Age acceleration; PhenoAge advancement, Phenotypic Age advancement;

Model 1: age (continuous), sex (female, male), race/ ethnicity (Mexican-American, other Hispanic, non-Hispanic white, non-Hispanic black, another race);

Model 2: age (continuous), sex (female, male), race/ ethnicity (Mexican-American, other Hispanic, non-Hispanic white, non-Hispanic black, other race), education level (less than high school, high school, more than high school), marital status (married/cohabitating, divorced/widowed/separated, never married), Poverty income ratio (<1.3, 1.3-3.5, >3.5). body mass index (<25.0 kg/m², 25.0-29.9 kg/m², ≥30.0 kg/m²), smoking status (never, former, current), heavy alcohol consumption (male ≥20g/day, female ≥10g/day), physical activity (less, moderate, vigorous), Type 2 Diabetes (Yes/no), hypertension (Yes/no) and cardiovascular diseases (Yes/no);

*: PhenoAge advancement is a binary variable, the results are shown as OR, 95%CI;

Investigation of α-Klotho is conducted exclusively in males and females aged 40 to 79 years;

The cobalamin insensitivity index estimated by homocysteine and cobalamin

Cobalamin insensitivity index based on homocysteine and cobalamin: a multiplicative term by homocysteine and cobalamin divided by 100

**Table S10. The Relationship between the cobalamin insensitivity index based on methylmalonic acid and cobalamin and biological aging**

|  | **Crude** | | **Model 1** | | **Model 2** | |
| --- | --- | --- | --- | --- | --- | --- |
|  | **β 95%CI** | ***P*** | **β 95%CI** | ***P*** | **β 95%CI** | ***P*** |
| **Cbl supplements, yes** |  |  |  |  |  |  |
| KDMAge | 7.86 (7.02 to 8.70) | P<0.001 | 1.38 (0.81 to 1.95) | P<0.001 | 1.39 (0.82 to 1.96) | P<0.001 |
| Phenotypic Age | 8.78 (7.63 to 9.92) | P<0.001 | 1.74 (0.54 to 2.94) | 0.01 | 1.73 (0.58 to 2.88) | P<0.001 |
| KDMAccel | 0.83 (0.30 to 1.37) | P<0.001 | 1.38 (0.81 to 1.95) | P<0.001 | 1.39 (0.82 to 1.96) | P<0.001 |
| PhenoAgeAccel | 1.76 (0.68 to 2.84) | P<0.001 | 1.74 (0.54 to 2.94) | 0.01 | 1.73 (0.58 to 2.88) | P<0.001 |
| Telomere length | -0.037 (-0.052 to -0.022) | P<0.001 | 0.003 (-0.012 to 0.018) | 0.698 | 0.002 (-0.013 to 0.017) | 0.776 |
| α-Klotho | 4.37 (-19.09 to 27.82) | 0.71 | 13.50 (-12.60 to 39.6) | 0.30 | 9.07 (-14.45 to 32.6) | 0.44 |
| PhenoAge advancement* | 0.97 (0.90 to 1.05) | 0.46 | 1.18 (1.09 to 1.29) | P<0.001 | 1.21 (1.10 to 1.33) | P<0.001 |
| **Cbl supplements, no** |  |  |  |  |  |  |
| KDMAge | 6.22 (5.48 to 6.97) | P<0.001 | 0.96 (0.66 to 1.25) | P<0.001 | 0.93 (0.67 to 1.18) | P<0.001 |
| Phenotypic Age | 7.09 (6.28 to 7.89) | P<0.001 | 1.29 (0.93 to 1.65) | P<0.001 | 1.22 (0.90 to 1.54) | P<0.001 |
| KDMAccel | 0.69 (0.42 to 0.97) | P<0.001 | 0.96 (0.66 to 1.25) | P<0.001 | 0.93 (0.67 to 1.18) | P<0.001 |
| PhenoAgeAccel | 1.56 (1.23 to 1.89) | P<0.001 | 1.29 (0.93 to 1.65) | P<0.001 | 1.22 (0.90 to 1.54) | P<0.001 |
| Telomere length | -0.033 (-0.047 to -0.02) | P<0.001 | 0 (-0.012 to 0.012) | 0.988 | -0.002 (-0.014 to 0.011) | 0.797 |
| α-Klotho | 21.48 (3.76 to 39.20) | 0.02 | 27.88 (9.34 to 46.42) | P<0.001 | 27.58 (10.19 to 44.98) | P<0.001 |
| PhenoAge advancement* | 1.06 (0.98 to 1.14) | 0.17 | 1.18 (1.09 to 1.29) | P<0.001 | 1.22 (1.12 to 1.34) | P<0.001 |
| **Dietary Cbl < 5.1 ug/day** |  |  |  |  |  |  |
| KDMAge | 7.62 (6.97 to 8.26) | P<0.001 | 1.02 (0.80 to 1.24) | P<0.001 | 0.98 (0.77 to 1.19) | P<0.001 |
| Phenotypic Age | 8.44 (7.72 to 9.16) | P<0.001 | 1.23 (0.88 to 1.58) | P<0.001 | 1.24 (0.9 to 1.57) | P<0.001 |
| KDMAccel | 0.58 (0.37 to 0.80) | P<0.001 | 1.02 (0.80 to 1.24) | P<0.001 | 0.98 (0.77 to 1.19) | P<0.001 |
| PhenoAgeAccel | 1.41 (1.08 to 1.75) | P<0.001 | 1.23 (0.88 to 1.58) | P<0.001 | 1.24 (0.90 to 1.57) | P<0.001 |
| Telomere length | -0.04 (-0.05 to -0.03) | P<0.001 | 0.003 (-0.008 to 0.014) | 0.584 | 0.002 (-0.009 to 0.013) | 0.709 |
| α-Klotho | 8.05 (-6.41 to 22.5) | 0.27 | 16.13 (0.33 to 31.94) | 0.046 | 14.09 (-0.96 to 29.14) | 0.07 |
| PhenoAge advancement* | 0.98 (0.91 to 1.06) | 0.64 | 1.15 (1.07 to 1.24) | P<0.001 | 1.18 (1.08 to 1.29) | P<0.001 |
| **Dietary Cbl ≥ 5.1 ug/day** |  |  |  |  |  |  |
| KDMAge | 6.47 (5.60 to 7.35) | P<0.001 | 1.17 (0.57 to 1.77) | P<0.001 | 1.25 (0.66 to 1.83) | P<0.001 |
| Phenotypic Age | 7.41 (6.08 to 8.74) | P<0.001 | 1.63 (0.39 to 2.86) | 0.01 | 1.64 (0.47 to 2.82) | 0.01 |
| KDMAccel | 0.79 (0.21 to 1.37) | 0.01 | 1.17 (0.57 to 1.77) | P<0.001 | 1.25 (0.66 to 1.83) | P<0.001 |
| PhenoAgeAccel | 1.74 (0.57 to 2.90) | P<0.001 | 1.63 (0.39 to 2.86) | 0.01 | 1.64 (0.47 to 2.82) | 0.01 |
| Telomere length | -0.034 (-0.049 to -0.02) | P<0.001 | -0.004 (-0.017 to 0.01) | 0.590 | -0.006 (-0.021 to 0.009) | 0.401 |
| α-Klotho | 22.91 (-0.77 to 46.59) | 0.06 | 30.47 (6.6 to 54.33) | 0.01 | 25.10 (0.07 to 50.12) | 0.05 |
| PhenoAge advancement* | 1.00 (0.91 to 1.11) | 0.94 | 1.17 (1.06 to 1.30) | P<0.001 | 1.24 (1.10 to 1.40) | P<0.001 |

Abbreviations: MMA, methylmalonic acid; Cbl, cobalamin; KDM, Klemera-Doubal method; KDMAccel, KDM Age acceleration; PhenoAgeAccel, Phenotypic Age acceleration; PhenoAge advancement, Phenotypic Age advancement;

Model 1: age (continuous), sex (female, male), race/ ethnicity (Mexican-American, other Hispanic, non-Hispanic white, non-Hispanic black, another race);

Model 2: age (continuous), sex (female, male), race/ ethnicity (Mexican-American, other Hispanic, non-Hispanic white, non-Hispanic black, other race), education level (less than high school, high school, more than high school), marital status (married/cohabitating, divorced/widowed/separated, never married), Poverty income ratio (<1.3, 1.3-3.5, >3.5). body mass index (<25.0 kg/m², 25.0-29.9 kg/m², ≥30.0 kg/m²), smoking status (never, former, current), heavy alcohol consumption (male ≥20g/day, female ≥10g/day), physical activity (less, moderate, vigorous), Type 2 Diabetes (Yes/no), hypertension (Yes/no) and cardiovascular diseases (Yes/no);

*: PhenoAge advancement is a binary variable, the results are shown as OR, 95%CI;

Investigation of α-Klotho is conducted exclusively in males and females aged 40 to 79 years;

Cobalamin insensitivity index estimated by Methylmalonic Acid and cobalamin;

Cobalamin insensitivity index based on MMA and cobalamin: a multiplicative term by Methylmalonic Acid and cobalamin divided by 100.

**Table S11. The Relationship between the cobalamin insensitivity index based on homocysteine and cobalamin and biological aging**

|  | **Crude** | | **Model 1** | | **Model 2** | |
| --- | --- | --- | --- | --- | --- | --- |
|  | **β 95%CI** | ***P*** | **β 95%CI** | ***P*** | **β 95%CI** | ***P*** |
| **Cbl supplements, yes** |  |  |  |  |  |  |
| KDMAge | 9.31 (8.59 to 10.03) | P<0.001 | 0.90 (0.52 to 1.28) | P<0.001 | 1.01 (0.66 to 1.35) | P<0.001 |
| Phenotypic Age | 9.90 (9.08 to 10.71) | P<0.001 | 0.50 (-0.06 to 1.06) | 0.08 | 0.58 (0.08 to 1.07) | 0.02 |
| KDMAccel | 0.33 (-0.03 to 0.68) | 0.07 | 0.90 (0.52 to 1.28) | P<0.001 | 1.01 (0.66 to 1.35) | P<0.001 |
| PhenoAgeAccel | 0.90 (0.43 to 1.38) | P<0.001 | 0.50 (-0.06 to 1.06) | 0.08 | 0.58 (0.08 to 1.07) | 0.02 |
| Telomere length | -0.037 (-0.051 to -0.024) | P<0.001 | 0.02 (0.006 to 0.033) | 0.006 | 0.018 (0.004 to 0.031) | 0.015 |
| PhenoAge advancement* | 0.89 (0.81 to 0.98) | 0.02 | 1.09 (0.99 to 1.21) | 0.09 | 1.15 (1.03 to 1.27) | 0.01 |
| **Cbl supplements, no** |  |  |  |  |  |  |
| KDMAge | 7.25 (6.55 to 7.94) | P<0.001 | 1.18 (0.84 to 1.52) | P<0.001 | 1.18 (0.88 to 1.49) | P<0.001 |
| Phenotypic Age | 8.37 (7.49 to 9.25) | P<0.001 | 1.64 (1.19 to 2.09) | P<0.001 | 1.50 (1.10 to 1.90) | P<0.001 |
| KDMAccel | 1.01 (0.69 to 1.34) | P<0.001 | 1.18 (0.84 to 1.52) | P<0.001 | 1.18 (0.88 to 1.49) | P<0.001 |
| PhenoAgeAccel | 2.15 (1.74 to 2.57) | P<0.001 | 1.64 (1.19 to 2.09) | P<0.001 | 1.50 (1.10 to 1.90) | P<0.001 |
| Telomere length | -0.04 (-0.06 to -0.02) | P<0.001 | -0.001 (-0.02 to 0.017) | 0.901 | -0.003 (-0.023 to 0.017) | 0.760 |
| PhenoAge advancement* | 1.15 (1.05 to 1.27) | P<0.001 | 1.25 (1.13 to 1.37) | P<0.001 | 1.32 (1.19 to 1.46) | P<0.001 |
| **Dietary Cbl < 5.1 ug/day** |  |  |  |  |  |  |
| KDMAge | 8.81 (8.22 to 9.4) | P<0.001 | 1.01 (0.77 to 1.25) | P<0.001 | 1.05 (0.81 to 1.28) | P<0.001 |
| Phenotypic Age | 9.67 (8.94 to 10.39) | P<0.001 | 0.89 (0.57 to 1.21) | P<0.001 | 0.93 (0.63 to 1.23) | P<0.001 |
| KDMAccel | 0.63 (0.40 to 0.85) | P<0.001 | 1.01 (0.77 to 1.25) | P<0.001 | 1.05 (0.81 to 1.28) | P<0.001 |
| PhenoAgeAccel | 1.46 (1.14 to 1.77) | P<0.001 | 0.89 (0.57 to 1.21) | P<0.001 | 0.93 (0.63 to 1.23) | P<0.001 |
| Telomere length | -0.044 (-0.06 to -0.028) | P<0.001 | 0.008 (-0.009 to 0.025) | 0.330 | 0.007 (-0.01 to 0.023) | 0.412 |
| PhenoAge advancement* | 1.01 (0.94 to 1.08) | 0.78 | 1.18 (1.09 to 1.29) | P<0.001 | 1.25 (1.14 to 1.36) | P<0.001 |
| **Dietary Cbl ≥ 5.1 ug/day** |  |  |  |  |  |  |
| KDMAge | 7.31 (6.54 to 8.09) | P<0.001 | 0.69 (0.39 to 0.99) | P<0.001 | 0.84 (0.56 to 1.12) | P<0.001 |
| Phenotypic Age | 8.18 (7.30 to 9.06) | P<0.001 | 0.75 (0.28 to 1.21) | P<0.001 | 0.80 (0.41 to 1.18) | P<0.001 |
| KDMAccel | 0.29 (0.00 to 0.58) | 0.05 | 0.69 (0.39 to 0.99) | P<0.001 | 0.84 (0.56 to 1.12) | P<0.001 |
| PhenoAgeAccel | 1.18 (0.75 to 1.61) | P<0.001 | 0.75 (0.28 to 1.21) | P<0.001 | 0.80 (0.41 to 1.18) | P<0.001 |
| Telomere length | -0.038 (-0.056 to -0.021) | P<0.001 | 0.004 (-0.014 to 0.023) | 0.649 | 0.001 (-0.021 to 0.022) | 0.956 |
| PhenoAge advancement* | 0.90 (0.83 to 0.97) | 0.01 | 1.05 (0.97 to 1.14) | 0.23 | 1.13 (1.03 to 1.23) | 0.01 |

Abbreviations: Hcy, homocysteine; Cbl, cobalamin; Dietary Cbl: Cobalamin intake from food; KDM, Klemera-Doubal method; KDMAccel, KDM Age acceleration; PhenoAgeAccel, Phenotypic Age acceleration; PhenoAge advancement, Phenotypic Age advancement;

Model 1: age (continuous), sex (female, male), race/ ethnicity (Mexican-American, other Hispanic, non-Hispanic white, non-Hispanic black, another race);

Model 2: age (continuous), sex (female, male), race/ ethnicity (Mexican-American, other Hispanic, non-Hispanic white, non-Hispanic black, other race), education level (less than high school, high school, more than high school), marital status (married/cohabitating, divorced/widowed/separated, never married), Poverty income ratio (<1.3, 1.3-3.5, >3.5). body mass index (<25.0 kg/m², 25.0-29.9 kg/m², ≥30.0 kg/m²), smoking status (never, former, current), heavy alcohol consumption (male ≥20g/day, female ≥10g/day), physical activity (less, moderate, vigorous), Type 2 Diabetes (Yes/no), hypertension (Yes/no) and cardiovascular diseases (Yes/no);

*: PhenoAge advancement is a binary variable, the results are shown as OR, 95%CI;

Investigation of α-Klotho is conducted exclusively in males and females aged 40 to 79 years;

The cobalamin insensitivity index estimated by homocysteine and cobalamin

Cobalamin insensitivity index based on Hcy and cobalamin: a multiplicative term by homocysteine and cobalamin divided by 100.

**Table S12. The relationship between cobalamin and its biomarkers and biological aging among participants under 80 years of age**

|  | **KDM Age** | **Phenotypic Age** | **KDMAccel** | **PhenoAgeAccel** | **telomere length** | **α-Klotho** | **PhenoAge adv*** |
| --- | --- | --- | --- | --- | --- | --- | --- |
|  | **β 95%CI** | **β 95%CI** | **β 95%CI** | **β 95%CI** | **β 95%CI** | **β 95%CI** | **β 95%CI** |
| Serum cobalamin, pg/ml | 0.39 (0.22 to 0.56) | 0.03 (-0.17 to 0.22) | 0.39 (0.22 to 0.56) | 0.03 (-0.17 to 0.22) | 0.004 (-0.008 to 0.015) | 23.18 (12.56 to 33.8) | 1.08 (1.02 to 1.15) |
| Dietary Cbl, ug/day | 0.24 (0.18 to 0.31) | 0.16 (0.06 to 0.25) | 0.24 (0.18 to 0.31) | 0.16 (0.06 to 0.25) | 0.004 (-0.001 to 0.010) | 5.73 (-4.39 to 15.86) | 1.12 (1.08 to 1.15) |
| Cobalamin supplments, % | -0.08 (-0.32 to 0.16) | 0.01 (-0.37 to 0.39) | -0.08 (-0.32 to 0.16) | 0.01 (-0.37 to 0.39) | -0.007 (-0.024 to 0.009) | -0.73 (-17.68 to 16.22) | 1.00 (0.89 to 1.12) |
| Methylmalonic acid, nmol/L | 1.05 (0.71 to 1.39) | 1.8 (1.14 to 2.46) | 1.05 (0.71 to 1.39) | 1.80 (1.14 to 2.46) | -0.003 (-0.017 to 0.011) | -4.02 (-16.29 to 8.25) | 1.20 (1.10 to 1.30) |
| Homocysteine, μmol/l | 1.25 (0.96 to 1.53) | 1.89 (1.43 to 2.35) | 1.25 (0.96 to 1.53) | 1.89 (1.43 to 2.35) | 0.006 (-0.016 to 0.028) | - | 1.24 (1.14 to 1.35) |
| Combination of MMA and Cbl |  |  |  |  |  |  |  |
| MMA_low_Cbl_low_ | Ref. | Ref. | Ref. | Ref. | Ref. | Ref. | 1.00 (Ref.) |
| MMA_low_Cbl_high_ | 0.21 (-0.04 to 0.46) | -0.1 (-0.36 to 0.15) | 0.21 (-0.04 to 0.46) | -0.1 (-0.36 to 0.15) | 0.003 (-0.012 to 0.018) | 37.16 (19.03 to 55.29) | 1.02 (0.88 to 1.18) |
| MMA_high_Cbl_low_ | 0.51 (0.04 to 0.98) | 1.66 (0.97 to 2.35) | 0.51 (0.04 to 0.98) | 1.66 (0.97 to 2.35) | -0.004 (-0.039 to 0.030) | 26.03 (-3.39 to 55.46) | 1.01 (0.83 to 1.24) |
| MMA_high_Cbl_high_ | 8.59 (6.00 to 11.19) | 11.42 (6.43 to 16.4) | 8.59 (6.00 to 11.19) | 11.42 (6.43 to 16.4) | -0.019 (-0.084 to 0.047) | -23.43 (-88.54 to 41.69) | 2.59 (1.58 to 4.24) |
| Combination of Hcy and Cbl |  |  |  |  |  |  |  |
| Hcy_low_Cbl_low_ | Ref. | Ref. | Ref. | Ref. | Ref. | - | 1.00 (Ref.) |
| Hcy_low_Cbl_high_ | 0.27 (0.07 to 0.47) | -0.05 (-0.29 to 0.19) | 0.27 (0.07 to 0.47) | -0.05 (-0.29 to 0.19) | 0.005 (-0.014 to 0.024) | - | 1.05 (0.94 to 1.17) |
| Hcy_high_Cbl_low_ | 0.94 (0.52 to 1.37) | 2.34 (1.84 to 2.85) | 0.94 (0.52 to 1.37) | 2.34 (1.84 to 2.85) | 0.010 (-0.028 to 0.048) | - | 1.14 (0.97 to 1.33) |
| Hcy_high_Cbl_high_ | 6.98 (5.38 to 8.58) | 8.26 (6.47 to 10.06) | 6.98 (5.38 to 8.58) | 8.26 (6.47 to 10.06) | -0.025 (-0.065 to 0.015) | - | 3.09 (2.14 to 4.48) |

Abbreviations: MMA, methylmalonic acid; Hcy, homocysteine; Cbl, cobalamin; Dietary Cbl: Cobalamin intake from food; KDM, Klemera-Doubal method; KDMAccel, KDM Age acceleration; PhenoAgeAccel, Phenotypic Age acceleration; PhenoAge adv, Phenotypic Age advancement;

Adjust for Model 2: age (continuous), sex (female, male), race/ ethnicity (Mexican-American, other Hispanic, non-Hispanic white, non-Hispanic black, other race), education level (less than high school, high school, more than high school), marital status (married/cohabitating, divorced/widowed/separated, never married), Poverty income ratio (<1.3, 1.3-3.5, >3.5). body mass index (<25.0 kg/m²), 25.0-29.9 kg/m², ≥30.0 kg/m²), smoking status (never, former, current), heavy alcohol consumption (male ≥20g/day, female ≥10g/day), physical activity (less, moderate, vigorous), Type 2 Diabetes (Yes/no), hypertension (Yes/no) and cardiovascular diseases (Yes/no).

*: PhenoAge advancement is a binary variable, the results are shown as OR, 95%CI

Investigation of α-Klotho is conducted exclusively in males and females aged 40 to 79 years

Combination of MMA or Hcy and cobalamin: Both indicators were categorized into high vs. low levels and combined into four groups according to the prespecified cutoff values (MMA >250 nmol/L or Hcy>12.1μmol/l and cobalamin >400 pg/mL)

**Table S13. The relationship between cobalamin and its biomarkers and** **biological aging stratified by sex**

|  | **KDMAccel** | | **PhenoAgeAccel** | | **PhenoAge advancement^*^** | |
| --- | --- | --- | --- | --- | --- | --- |
|  | **β 95%CI** | ***P*** | **β 95%CI** | ***P*** | **OR 95%CI** | ***P*** |
| **Female, (11,812)** |  |  |  |  |  |  |
| Serum cobalamin, pg/ml | 0.26 (0.05 to 0.48) | 0.02 | -0.12 (-0.36 to 0.13) | 0.33 | 1.04 (0.97 to 1.12) | 0.26 |
| Cobalamin intake from food, ug/day | 0.268 (0.18 to 0.36) | P<0.001 | 0.22 (0.12 to 0.33) | P<0.001 | 1.13 (1.07 to 1.19) | P<0.001 |
| Cobalamin supplements, % | 0.25 (-0.06 to 0.56) | 0.11 | 0.31 (-0.10 to 0.72) | 0.14 | 1.15 (0.98 to 1.35) | 0.082 |
| Methylmalonic acid, nmol/L | 0.92 (0.70 to 1.14) | P<0.001 | 1.55 (1.05 to 2.04) | P<0.001 | 1.28 (1.17 to 1.40) | P<0.001 |
| Homocysteine, μmol/l | 1.33 (1.01 to 1.65) | P<0.001 | 1.92 (1.42 to 2.43) | P<0.001 | 1.32 (1.17 to 1.50) | P<0.001 |
| Combination of MMA and Cbl |  |  |  |  |  |  |
| MMA_low_Cbll_ow_ | Ref. |  | Ref. |  | 1.00 (Ref.) |  |
| MMA_low_Cbl_high_ | 0.08 (-0.21 to 0.37) | 0.59 | -0.15 (-0.44 to 0.15) | 0.32 | 1.01 (0.87 to 1.17) | 0.91 |
| MMA_high_Cbl_low_ | 0.78 (0.19 to 1.37) | 0.01 | 1.64 (0.93 to 2.36) | P<0.001 | 1.21 (0.95 to 1.54) | 0.11 |
| MMA_high_Cbl_high_ | 6.97 (4.41 to 9.54) | P<0.001 | 9.1 (4.58 to 13.61) | P<0.001 | 2.67 (1.59 to 4.50) | P<0.001 |
| Combination of Hcy and Cbl |  |  |  |  |  |  |
| Hcy_low_Cbl_low_ | Ref. |  | Ref. |  | 1.00 (Ref.) |  |
| Hcy_low_Cbl_high_ | 0.18 (-0.04 to 0.41) | 0.11 | 0.00 (-0.28 to 0.27) | 0.99 | 1.05 (0.93 to 1.18) | 0.42 |
| Hcy_high_Cbl_low_ | 1.6 (0.99 to 2.21) | P<0.001 | 3.08 (2.36 to 3.80) | P<0.001 | 1.57 (1.22 to 2.02) | P<0.001 |
| Hcy_high_Cbl_high_ | 7.81 (6.2 to 9.41) | P<0.001 | 8.24 (6.34 to 10.15) | P<0.001 | 5.92 (3.65 to 9.62) | P<0.001 |
| **Male, (11,000)** |  |  |  |  |  |  |
| Serum cobalamin, pg/ml | 0.67 (0.44 to 0.90) | P<0.001 | 0.51 (0.25 to 0.77) | P<0.001 | 1.15 (1.04 to 1.27) | 0.01 |
| Cobalamin intake from food, ug/day | 0.20 (0.11 to 0.30) | P<0.001 | 0.09 (-0.03 to 0.22) | 0.14 | 1.09 (1.04 to 1.15) | P<0.001 |
| Cobalamin supplements, % | -0.35 (-0.65 to -0.05) | 0.02 | -0.34 (-0.86 to 0.17) | 0.18 | 0.89 (0.77 to 1.02) | 0.101 |
| Methylmalonic acid, nmol/L | 1.33 (0.74 to 1.92) | P<0.001 | 2.19 (1.10 to 3.28) | P<0.001 | 1.20 (1.08 to 1.34) | P<0.001 |
| Homocysteine, μmol/l | 1.57 (1.14 to 1.99) | P<0.001 | 2.56 (1.93 to 3.18) | P<0.001 | 1.27 (1.12 to 1.44) | P<0.001 |
| Combination of MMA and Cbl |  |  |  |  |  |  |
| MMA_low_Cbll_ow_ | Ref. |  | Ref. |  | 1.00 (Ref.) |  |
| MMA_low_Cbl_high_ | 0.50 (0.13 to 0.86) | 0.01 | 0.24 (-0.09 to 0.56) | 0.15 | 1.07 (0.87 to 1.33) | 0.51 |
| MMA_high_Cbl_low_ | 0.95 (0.23 to 1.68) | 0.01 | 2.01 (1.01 to 3.01) | P<0.001 | 1.15 (0.87 to 1.52) | 0.32 |
| MMA_high_Cbl_high_ | 9.14 (4.95 to 13.33) | P<0.001 | 12.8 (5.13 to 20.46) | P<0.001 | 2.81 (1.53 to 5.17) | P<0.001 |
| Combination of Hcy and Cbl |  |  |  |  |  |  |
| Hcy_low_Cbl_low_ | Ref. |  | Ref. |  | 1.00 (Ref.) |  |
| Hcy_low_Cbl_high_ | 0.47 (0.16 to 0.78) | P<0.001 | 0.21 (-0.18 to 0.61) | 0.28 | 1.07 (0.90 to 1.28) | 0.42 |
| Hcy_high_Cbl_low_ | 1.05 (0.58 to 1.53) | P<0.001 | 2.08 (1.47 to 2.69) | P<0.001 | 1.14 (0.93 to 1.40) | 0.19 |
| Hcy_high_Cbl_high_ | 6.41 (4.47 to 8.34) | P<0.001 | 7.88 (5.73 to 10.03) | P<0.001 | 2.59 (1.74 to 3.85) | P<0.001 |

Abbreviations: MMA, methylmalonic acid; Hcy, homocysteine; Cbl, cobalamin; KDM, Klemera-Doubal method; KDMAccel, KDM Age acceleration; PhenoAgeAccel, Phenotypic Age acceleration; PhenoAge advancement, Phenotypic Age advancement; OR, odds ratio; CI, confidence interval;

Adjust for Model 2: age (continuous), sex (female, male), race/ ethnicity (Mexican-American, other Hispanic, non-Hispanic white, non-Hispanic black, another race), education level (less than high school, high school, more than high school), marital status (married/cohabitating, divorced/widowed/separated, never married), Poverty income ratio (<1.3, 1.3-3.5, >3.5). body mass index (<25.0 kg/m²), 25.0-29.9 kg/m², ≥30.0 kg/m²), smoking status (never, former, current), heavy alcohol consumption (male ≥20g/day, female ≥10g/day), physical activity (less, moderate, vigorous), Type 2 Diabetes (Yes/no), hypertension (Yes/no) and cardiovascular diseases (Yes/no).

*: PhenoAge advancement is a binary variable, the results are shown as OR, 95%CI

Investigation of α-Klotho is conducted exclusively in males and females aged 40 to 79 years

Combination of MMA or Hcy and cobalamin: Both indicators were categorized into high vs. low levels and combined into four groups according to the prespecified cutoff values (MMA >250 nmol/L or Hcy>12.1μmol/l and cobalamin >400 pg/mL)

**Table S14. The relationship between cobalamin and its biomarkers and biological aging stratified by age**

|  | **KDMAccel** | | **PhenoAgeAccel** | | **PhenoAge advancement*** | | |
| --- | --- | --- | --- | --- | --- | --- | --- |
|  | **β 95%CI** | ***P*** | **β 95%CI** | ***P*** | **OR 95%CI** | | ***P*** |
| **20-40 years (n=6,337)** |  |  |  |  |  | |  |
| Serum cobalamin, pg/ml | 0.17 (-0.07 to 0.40) | 0.16 | -0.02 (-0.34 to 0.3) | 0.93 | 0.97 (0.87 to 1.08) | | 0.58 |
| Cobalamin intake from food, ug/day | 0.29 (0.20 to 0.38) | P<0.001 | 0.20 (0.06 to 0.34) | 0.01 | 1.14 (1.07 to 1.21) | | P<0.001 |
| Cobalamin supplements, nmol/L | 0.03 (-0.27 to 0.33) | 0.86 | 0.51 (0.07 to 0.96) | 0.02 | 1.09 (0.91 to 1.31) | | 0.34 |
| Methylmalonic acid, nmol/L | 0.37 (0.05 to 0.68) | 0.02 | 0.99 (0.50 to 1.48) | P<0.001 | 1.08 (0.93 to 1.25) | | 0.29 |
| Homocysteine, μmol/l | 0.41 (-0.03 to 0.85) | 0.06 | 0.83 (0.14 to 1.51) | 0.02 | 0.99 (0.85 to 1.15) | | 0.86 |
| Combination of MMA and Cbl |  |  |  |  |  | |  |
| MMA_low_Cbll_ow_ | Ref. |  |  |  | 1.00 (Ref.) | |  |
| MMA_low_Cbl_high_ | 0.08 (-0.25 to 0.40) | 0.64 | -0.02 (-0.46 to 0.42) | 0.92 | 1.00 (0.84 to 1.19) | | 1.00 |
| MMA_high_Cbl_low_ | -0.15 (-1.21 to 0.91) | 0.78 | 0.64 (-0.74 to 2.03) | 0.35 | 0.91 (0.64 to 1.28) | | 0.57 |
| MMA_high_Cbl_high_ | 4.06 (0.46 to 7.65) | 0.03 | 6.81 (0.61 to 13.01) | 0.03 | 1.76 (0.73 to 4.22) | | 0.20 |
| Combination of Hcy and Cbl |  |  |  |  |  | |  |
| Hcy_low_Cbl_low_ | Ref. |  | Ref. |  | 1.00 (Ref.) | |  |
| Hcy_low_Cbl_high_ | 0.11 (-0.15 to 0.37) | 0.41 | -0.1 (-0.46 to 0.26) | 0.57 | 0.99 (0.87 to 1.13) | | 0.87 |
| Hcy_high_Cbl_low_ | 0.52 (-0.60 to 1.64) | 0.36 | 1.77 (0.37 to 3.18) | 0.01 | 0.77 (0.54 to 1.10) | | 0.14 |
| Hcy_high_Cbl_high_ | 3.68 (0.62 to 6.75) | 0.02 | 8.47 (4.08 to 12.86) | P<0.001 | 1.02 (0.42 to 2.46) | | 0.97 |
| **40-60 years (n=8,187)** |  |  |  |  |  | |  |
| Serum cobalamin, pg/ml | 0.43 (0.18 to 0.68) | P<0.001 | 0.14 (-0.24 to 0.52) | 0.47 | 1.15 (1.05 to 1.26) | | P<0.001 |
| Cobalamin intake from food, ug/day | 0.22 (0.11 to 0.32) | P<0.001 | 0.20 (0.06 to 0.33) | 0.01 | 1.13 (1.06 to 1.19) | | P<0.001 |
| Cobalamin supplements, nmol/L | -0.06 (-0.51 to 0.38) | 0.77 | 0.03 (-0.71 to 0.76) | 0.94 | 0.98 (0.83 to 1.15) | | 0.76 |
| Methylmalonic acid, nmol/L | 1.18 (0.36 to 2.00) | 0.01 | 2.10 (0.55 to 3.65) | 0.01 | 1.15 (0.99 to 1.33) | | 0.07 |
| Homocysteine, μmol/l | 0.71 (0.29 to 1.12) | P<0.001 | 1.79 (1.17 to 2.40) | P<0.001 | 1.12 (0.99 to 1.27) | | 0.07 |
| Combination of MMA and Cbl |  |  |  |  |  | |  |
| MMA_low_Cbll_ow_ | Ref. |  | Ref. |  | 1.00 (Ref.) | |  |
| MMA_low_Cbl_high_ | 0.27 (-0.11 to 0.65) | 0.16 | -0.1 (-0.57 to 0.37) | 0.68 | 0.99 (0.79 to 1.24) | | 0.9 |
| MMA_high_Cbl_low_ | -0.08 (-1.09 to 0.93) | 0.87 | 0.85 (-0.49 to 2.19) | 0.21 | 0.67 (0.41 to 1.09) | | 0.11 |
| MMA_high_Cbl_high_ | 9.17 (2.84 to 15.49) | 0.01 | 15.61 (2.45 to 28.76) | 0.02 | 2.31 (0.99 to 5.36) | | 0.05 |
| Combination of Hcy and Cbl |  |  |  |  |  | |  |
| Hcy_low_Cbl_low_ | Ref. |  | Ref. |  | 1.00 (Ref.) | |  |
| Hcy_low_Cbl_high_ | 0.35 (0.01 to 0.69) | 0.05 | 0.06 (-0.42 to 0.53) | 0.81 | 1.06 (0.89 to 1.27) | | 0.49 |
| Hcy_high_Cbl_low_ | 0.07 (-0.46 to 0.61) | 0.78 | 1.42 (0.78 to 2.07) | P<0.001 | 0.96 (0.75 to 1.24) | | 0.77 |
| Hcy_high_Cbl_high_ | 5.81 (2.54 to 9.08) | P<0.001 | 7.12 (4.01 to 10.24) | P<0.001 | 2.78 (1.50 to 5.16) | | P<0.001 |
| **≥60 years (n=8,288)** |  |  |  |  | |  |  |
| Serum cobalamin, pg/ml | 0.62 (0.26 to 0.98) | P<0.001 | 0.2 (-0.23 to 0.63) | 0.35 | | 1.13 (1.01 to 1.27) | 0.03 |
| Cobalamin intake from food, ug/day | 0.15 (-0.01 to 0.30) | 0.07 | -0.01 (-0.21 to 0.18) | 0.88 | | 1.03 (0.96 to 1.11) | 0.41 |
| Cobalamin supplements, nmol/L | -0.14 (-0.61 to 0.33) | 0.55 | -0.68 (-1.28 to -0.08) | 0.03 | | 0.90 (0.76 to 1.08) | 0.26 |
| Methylmalonic acid, nmol/L | 2.03 (1.63 to 2.42) | P<0.001 | 2.60 (2.01 to 3.18) | P<0.001 | | 1.65 (1.42 to 1.90) | P<0.001 |
| Homocysteine, μmol/l | 4.23 (3.58 to 4.88) | P<0.001 | 4.83 (4.17 to 5.49) | P<0.001 | | 3.06 (2.48 to 3.77) | P<0.001 |
| Combination of MMA and Cbl |  |  |  |  | |  |  |
| MMA_low_Cbll_ow_ | Ref. |  | Ref. |  | | 1.00 (Ref.) |  |
| MMA_low_Cbl_high_ | 0.63 (0.19 to 1.07) | 0.01 | 0.36 (-0.18 to 0.91) | 0.19 | | 1.27 (1.02 to 1.59) | 0.04 |
| MMA_high_Cbl_low_ | 2.44 (1.74 to 3.13) | P<0.001 | 3.25 (2.33 to 4.18) | P<0.001 | | 2.20 (1.68 to 2.90) | P<0.001 |
| MMA_high_Cbl_high_ | 9.04 (6.45 to 11.63) | P<0.001 | 9.48 (6.17 to 12.80) | P<0.001 | | 3.69 (2.27 to 6.00) | P<0.001 |
| Combination of Hcy and Cbl |  |  |  |  | |  |  |
| Hcy_low_Cbl_low_ | Ref. |  | Ref. |  | | 1.00 (Ref.) |  |
| Hcy_low_Cbl_high_ | 0.57 (0.21 to 0.93) | P<0.001 | 0.41 (-0.09 to 0.90) | 0.11 | | 1.28 (1.06 to 1.55) | 0.01 |
| Hcy_high_Cbl_low_ | 2.87 (2.37 to 3.36) | P<0.001 | 3.65 (3.04 to 4.25) | P<0.001 | | 2.30 (1.89 to 2.81) | P<0.001 |
| Hcy_high_Cbl_high_ | 8.79 (7.24 to 10.34) | P<0.001 | 8.45 (6.39 to 10.51) | P<0.001 | | 6.01 (4.44 to 8.13) | P<0.001 |

Abbreviations: MMA, methylmalonic acid; Hcy, homocysteine; Cbl, cobalamin; KDM, Klemera-Doubal method; KDMAccel, KDM Age acceleration; PhenoAgeAccel, Phenotypic Age acceleration; PhenoAge advancement, Phenotypic Age advancement; OR, odds ratio; CI, confidence interval;

Adjust for Model 2: age (continuous), sex (female, male), race/ ethnicity (Mexican-American, other Hispanic, non-Hispanic white, non-Hispanic black, other race), education level (less than high school, high school, more than high school), marital status (married/cohabitating, divorced/widowed/separated, never married), Poverty income ratio (<1.3, 1.3-3.5, >3.5). body mass index (<25.0 kg/m²), 25.0-29.9 kg/m², ≥30.0 kg/m²), smoking status (never, former, current), heavy alcohol consumption (male ≥20g/day, female ≥10g/day), physical activity (less, moderate, vigorous), Type 2 Diabetes (Yes/no), hypertension (Yes/no) and cardiovascular diseases (Yes/no).

*: PhenoAge advancement is a binary variable, the results are shown as OR, 95%CI

Investigation of α-Klotho is conducted exclusively in males and females aged 40 to 79 years

Combination of MMA or Hcy and cobalamin: Both indicators were categorized into high vs. low levels and combined into four groups according to the prespecified cutoff values (MMA >250 nmol/L or Hcy>12.1μmol/l and cobalamin >400 pg/mL)

**Supplementary Methods**

1. **KDM Biological Age and KDMAccel**

$$KDM Age=\frac{\sum_{i=1}^{n} \left( x_{i}-q_{i} \right)\frac{k_{i}}{s_{i}^{2}}+\frac{Chronological Age}{s_{BA}^{2}}}{\sum_{i=1}^{n} \left( \frac{k_{i}}{s_{i}} \right)^{2}+\frac{1}{s_{BA}^{2}}}$$

$$KDMAccel=KDM Age-Chronological Age$$

Where *x* is the value of biomarker *i* measured for an individual. For each biomarker *i*, the parameters *k*, *q*, and *s* are estimated from a regression of chronological age on the biomarker in the reference sample. The *q*, *k*, and *s* are the regression intercept, slope, and root mean squared error, respectively. *s_BA_* is a scaling factor equal to the square root of the variance in chronological age explained by the biomarker set in the reference sample.

**2. Phenotypic Age and PhenoAgeAccel**

$$Phenotypic Age=141.50+\frac{\ln\left[ -0.00553\times ln \left( \exp\left( \frac{-1.51714\times\exp\left( xb \right)}{0.0076927} \right) \right) \right]}{0.09165}$$

$$PhenoAgeAccel=Phenotypic Age-Chronological Age$$

Where:

$$xb = - 19.907 - 0.0336 \times Albumin + 0.0095 \times Creatinine + 0.1953 \times Glucose + 0.0954 \times LnCRP-0.0120 \times Lymphocyte Percent + 0.0268 \times Mean Cell Volume + 0.3306 \times Red Cell Distribution Width + 0.00188 \times Alkaline Phosphatase + 0.0554 \times White Blood Cell Count + 0.0804 \times Chronological Age$$
